# Whole Genome Sequencing and Spatial Analysis Identifies Recent Tuberculosis Transmission Hotspots in Ghana

**DOI:** 10.3389/fmed.2020.00161

**Published:** 2020-05-19

**Authors:** Prince Asare, Isaac Darko Otchere, Edmund Bedeley, Daniela Brites, Chloé Loiseau, Nyonuku Akosua Baddoo, Adwoa Asante-Poku, Stephen Osei-Wusu, Diana Ahu Prah, Sonia Borrell, Miriam Reinhard, Audrey Forson, Kwadwo Ansah Koram, Sebastien Gagneux, Dorothy Yeboah-Manu

**Affiliations:** ^1^Noguchi Memorial Institute for Medical Research, College of Health Sciences, University of Ghana, Accra, Ghana; ^2^West African Centre for Cell Biology of Infectious Pathogens, University of Ghana, Accra, Ghana; ^3^Department of Biochemistry, Cell and Molecular Biology, University of Ghana, Accra, Ghana; ^4^Department of Medical Parasitology and Infection Biology, Swiss Tropical and Public Health Institute, Basel, Switzerland; ^5^University of Basel, Basel, Switzerland; ^6^Department of Chest Diseases, Korle-Bu Teaching Hospital, Korle-Bu, Accra, Ghana

**Keywords:** *Mycobacterium tuberculosis*, *Mycobacterium africanum*, molecular epidemiology, whole genome sequence, recent transmission, cluster

## Abstract

Whole genome sequencing (WGS) is progressively being used to investigate the transmission dynamics of *Mycobacterium tuberculosis* complex (MTBC). We used WGS analysis to resolve traditional genotype clusters and explored the spatial distribution of confirmed recent transmission clusters. Bacterial genomes from a total of 452 MTBC isolates belonging to large traditional clusters from a population-based study spanning July 2012 and December 2015 were obtained through short read next-generation sequencing using the illumina HiSeq2500 platform. We performed clustering and spatial analysis using specified R packages and ArcGIS. Of the 452 traditional genotype clustered genomes, 314 (69.5%) were confirmed clusters with a median cluster size of 7.5 genomes and an interquartile range of 4–12. Recent tuberculosis (TB) transmission was estimated as 24.7%. We confirmed the wide spread of a Cameroon sub-lineage clone with a cluster size of 78 genomes predominantly from the Ablekuma sub-district of Accra metropolis. More importantly, we identified a recent transmission cluster associated with isoniazid resistance belonging to the Ghana sub-lineage of lineage 4. WGS was useful in detecting unsuspected outbreaks; hence, we recommend its use not only as a research tool but as a surveillance tool to aid in providing the necessary guided steps to track, monitor, and control TB.

## Introduction

Tuberculosis (TB), a contagious disease of antiquity, affects millions of people annually (1). In 1993, the World Health Organization (WHO) declared it a global health emergency, hence calling for more resources and studies for effective control of the disease. In 2017 alone, an estimated 10 million new TB cases with 1.6 million deaths were reported globally, making TB the number one infectious disease killer and one of the top 10 killer diseases (1). TB is caused by a group of closely related acid-fast bacilli called *Mycobacterium tuberculosis* complex (MTBC). TB in humans is caused mainly by *M. tuberculosis sensu stricto* (MTBss) and *M. africanum* (MAF), which are further divided into seven lineages (L): MTBss subdivided into L1–L4 and L7, and MAF L5 and L6 (2, 3).

The global TB control strategy aims at having a TB-free world by attaining zero deaths, disease, and suffering due to TB (4). One of the activities to achieve this is to investigate the transmission dynamics of the disease to understand risk factors leading to occurrence of the disease within distinct population for design of appropriate preventive interventions. The study of the spread of these TB lineages has been made possible through molecular epidemiology (molepi) studies (5). The traditional molepi tools including IS*6110* DNA fingerprinting, spacer oligonucleotide typing (spoligotyping), and mycobacteria interspersed repetitive-unit–variable-number tandem repeat (MIRU-VNTR) typing have been used extensively in previous studies and found to have varying discriminatory power (6, 7). However, whole genome sequencing (WGS) analysis is considered the ultimate for strain typing and confirmation of strain clusters (7–9).

In a previous population-based study, we used the combined resolution power of MIRU-VNTR typing and spoligotyping (MIRU/Spoligo) for strain differentiation followed with clustering analysis to estimate the extent of recent transmission in Ghana (10). We estimated a high recent transmission rate of 41.2% and found 53.1% of all isolates belonging to one of 276 clusters. Yet, it has been indicated that the combined resolution of spoligotyping and MIRU-VNTR may not be enough to distinguish between very closely related strains resulting from recent transmission (7). Consequently, in this current study, we used a WGS approach to further resolve large MIRU/Spoligo defined TB clusters (referred to as traditional clusters) and explore epidemiological factors including their spatial distribution.

## Materials and Methods

### Study Design and Population

This study involved a retrospective analysis of selected isolates obtained from a population-based study conducted from July 2012 to December 2015 and sampled within two administrative districts in Ghana: Accra Metropolitan Assembly (AMA) and East Mamprusi District (MamE). All isolates were obtained from pulmonary TB cases with informed consent from all participants. Within the population-based prospective study, sputum samples were collected from consecutive clinically diagnosed pulmonary TB patients reporting to 12 selected health facilities within an urban setting (AMA) and a rural setting (MamE). The methods that were used for sputum sampling during the population-based study conformed to WHO guidelines (two sputa per patient). We defined a pulmonary TB case as any individual with a suspected case of TB that was confirmed both clinically and bacteriologically. Detailed demographic and epidemiological data were obtained from consented participants.

Further description of the study locale and participant data are provided elsewhere (5, 10).

### Isolate Selection, DNA Extraction, and WGS

The isolate collection for the analysis was a convenient sample of all cases belonging to 40 large clusters (cluster size > 5) comprising 473 isolates from our previous study (10). Every manipulation of live MTBC bacilli was done in the biosafety level 3 facility of the NMIMR. As a recap, a cluster was defined as two or more isolates (same strain) that share an indistinguishable spoligotype and 15 locus MIRU-VNTR allelic pattern, but allowing for one missing allelic data at any one of the *difficult-to-amplify* MIRU loci (VNTR 2,163, 3,690, and 4,156), following which we categorized the size of a cluster using the total number of isolates into categories of small (2 isolates), medium (3–5 isolates), large (6–20 isolates), and very large (>20 isolates). For this current study, only large and very large clusters belonging to the three most dominant MTBC lineages (L4, L5, and L6) in Ghana were considered for analysis. All isolates have been previously characterized including drug susceptibility to isoniazid and rifampicin using standard phenotypic and genotypic techniques (5, 10). DNA extraction was performed using a modified cetyl trimethyl ammonium bromide (CTAB) protocol as previously described (11). The only amendment to our previous extraction protocol was that, to obtain enough intact (non-fragmented) genomic DNA (gDNA), bacteria cells were heat inactivated at 80°C (instead of 95°C) for 30 min in cell lysis buffer. Heat inactivating the bacterial cells at 95°C rather produces a lot of fragmented gDNA, which is not ideal for obtaining a quality sequencing output. Illumina sequencing libraries were prepared using NEBNEXT ULTRA II FS DNA library preparation kit (New England Biolabs) and multiplex paired-end (or in special cases single-end) sequenced at the Genomics Facility of the University of Basel using the illumina HiSeq2500 NGS platform (Illumina, San Diego, CA, United States) with raw read sequence lengths of either 101, 125, or 126 nucleotides (nt). Information on raw sequence data (BioProject ID: PRJNA616081) are provided in Supplementary Table 1.

### Whole Genome Sequence Analysis and Variance Calling

The raw fastq illumina reads were trimmed of illumina adaptor and low-quality reads using Trimmomatic v 0.33 with a sliding window of 5:20 (12). We dropped all reads with read length <20 nt and employed the mem algorithm in BWA v0.7.13 (13) to align the filtered reads to a reconstructed MTBC ancestral sequence obtained from a previous report (14). The chromosome coordinates and the annotation used was based on the genome of the laboratory reference strain *M. tuberculosis* H37Rv (NC_000962.3). We excluded also duplicated reads after marking with the Mark Duplicate module of Picard v2.9.1 (https://github.com/broadinstitute/picard). Single-nucleotide polymorphisms (SNPs) were called with mpileup implemented in Samtools v1.2 (15) and VarScan v2.4.1 (16). We used a quality threshold score of 20 for both minimum mapping quality and minimum base quality. Sample SNPs were called using the majority allele (SNPs were considered to have reached fixation within an isolate with a minimum frequency of 90%) in positions supported by at least seven fold coverage; on the other hand, the ancestor state was called when the SNP within-isolate frequency was ≤ 10%; otherwise, we classified them as indeterminate. We classified a genome as a possible mixed infection or contaminated if it had more than 120 heterogeneous base calls. All SNPs were annotated using snpEff v4.11 (17) with H37Rv reference annotation (NC_000962.3). We excluded genome positions in highly repetitive and variable regions (PE/PPE genes), phages, insertion sequences, and regions with at least 50-bp identities to other regions in the genome (18). After all the filtering steps, we also additionally excluded genomes with average coverage lower than 15 ×, leaving 452 genomes for subsequent analysis. The mean coverage for all the 452 genomes was 77 × with a standard deviation of 27 × (Supplementary Table 1).

### Phylogenetic Analysis

All 452 genomes that passed the filtering steps were used to generate a multifasta alignment file containing only polymorphic sites using customized python scripts. A position was considered polymorphic if at least one genome had an SNP at that position. We excluded genome positions with >10% missing calls. Both the GTR-GAMMA and GTR-CAT models with 1,000 rapid bootstrap inferences followed by a thorough maximum-likelihood search performed in CIPRES (19) were used to infer a maximum likelihood phylogenetic tree using the MPI parallel version of RaxML v8.2.3 (20) on the multi-fasta alignment file. Phylogenetic trees constructed using the GTR-GAMMA model did not produce any substantially different topologies and did not affect clustering analysis compared to the GTR-CAT model; consequently, we resorted to using GTR-CAT since results are produced faster. The best-scoring maximum likelihood topology trees generated were rooted on *M. canettii* as outgroup. Phylogenetic trees were plotted and annotated using the ggtree package (21, 22) and graphics enhanced using ggplot2 (23) all implemented in R version 3.6.0 (24) (http://cran.r-project.org/). We calculated pairwise SNP distances between genomes using the ape package (25) implemented in R version 3.6.0 (24).

### Cluster Definition and Analysis

Clustering analysis was based on the assumption that strains with the same DNA fingerprint may be epidemiologically linked and associated with recent TB transmission (26). Only one genome per participant was included in the analysis. Based on proposed SNP thresholds from various studies, three genomic cluster definitions were explored; a cutoff at 5 SNPs (9), 10 SNPs (27), and 12 SNPs (28, 29). Using the multi-fasta file and the cluster package (30) implemented in R version 3.6.0 (24), we set the three thresholds of 5, 10, and 12 and generated separate datasets containing a list of clusters per SNP threshold specified. We performed further downstream analysis on selected clustered cases after sticking to a threshold of 10 SNPs. The size of a cluster was defined using the total number of genomes in the cluster classified into categories of small (2 genomes), medium (3–5 genomes), and large (>5 genomes). The recent TB transmission rate and population size used for clustering analysis was estimated using the *n* – 1 formula described by Glynn et al. (31) (see Supplementary Data) (31).

### Within-Host Micro-Evolution of Longitudinal Isolates

To help set a threshold for defining genomic cluster, we performed a within-host microevolution analysis using genomes from cases with multiple TB episodes (longitudinal isolates). In addition to the clustered cases, WGS of three randomly selected MTBC isolate pairs obtained from three longitudinal cases were carried out using the protocols described above. Isolates from these three cases were chosen because they belong to the three most dominant lineage/sub-lineages found in Ghana being Cameroon, Ghana, and MAF West African 1. These isolates were investigated for within-host micro-evolution by calculating pairwise SNP distances between each pair of sequence from the same case using MEGA v10.0.5 (32).

### Data Management and Analysis

We included in our analysis both genomic and epidemiological data. Analysis that involved statistical inferences was carried out using the Stata statistical package version 14.2 (Stata Corp., College Station, TX, USA). The GIS coordinates of the participants' self-reported district of residence were used to construct a spatial representation of the MTBC isolates using R version 3.6.0 (24) and the ArcMap employed in ArcGIS (Economic and Social Research Institute, version 10.1) (33). The GIS coordinate information was combined with the genomic, epidemiological, and other demographic data to analyze risk factors for clustering.

## Results

### Characteristics of the Study Population

**Figure 1:**
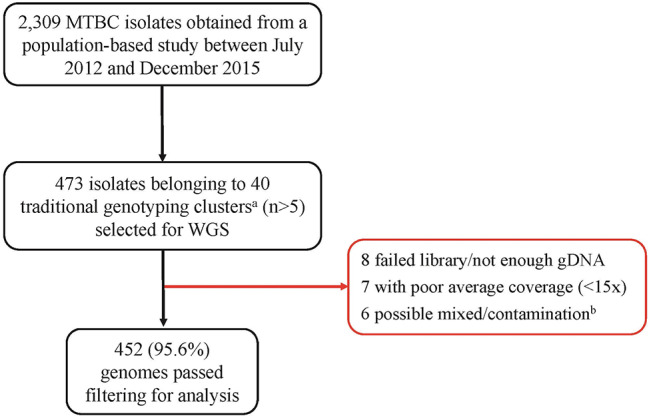
Pipeline for sample selection and whole genome sequencing. MTBC, *Mycobacterium tuberculosis* complex; WGS, Whole genome sequencing. ^a^We defined traditional genotyping clusters as previously described clusters identified using mycobacterial interspersed repetitive-unit–variable-number tandem-repeat analysis and spoligotyping tools. ^b^Genomes with heterozygous SNPs >120 were classified as possible mixed infection or contamination and hence removed from further analysis.

Out of the 473 isolates, each from a single case, 452 (95.6%) were passed for downstream analysis (Figure 1). Of the passed genomes, 71.4% (319/447) and 28.6% (128/447) of the infected participants were from males and females, respectively, with a median age of 35 years (range, 27 to 45 years). Five participants had no record of gender. A large proportion of the participants (73.6%, 315/428) had a sputum-smear microscopy grade of at least 2+.

### SNP Threshold Selection and Clustering Analysis

**Figure 2:**
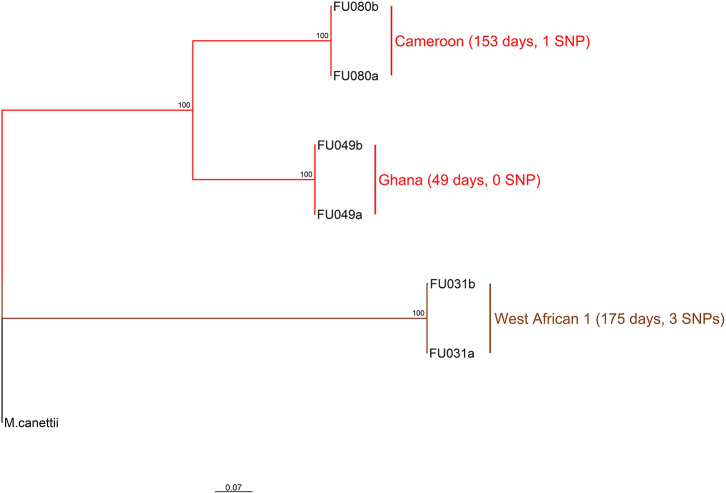
Phylogenetic relationship of six *M. tuberculosis* complex longitudinal isolates obtained from three participants. The two major branches constitute the top two MTBC lineages found in Ghana and color coded as red for lineage 4 and brown for lineage 5. Lineage 4 is further split into the dominant Cameroon and Ghana sub-lineage. The sub-lineages, days between sampling for each participant as well as the number of SNPs observed between sample pairs are annotated on the tree rooted on *M. canettii*. This analysis involved seven nucleotide sequences. All ambiguous positions were removed for each sequence pair (pairwise deletion option). There was a total of 2104 positions in the final dataset. Evolutionary analyses were conducted in MEGA v10.0.5 (32).

Three longitudinal TB cases were randomly chosen for within-host micro-evolution analysis. The three cases had two isolates each, belonging to the Cameroon (FU080), Ghana (FU049), and MAF West African 1 (FU031) genotypes. All three cases received the same set of anti-TB drugs (isoniazid, rifampicin, ethambutol, and pyrazinamide). Case FU080 was male, 39 years of age, diagnosed with a sputum-smear microscopy grade of 2+, and the follow-up sample was taken at month 5 (153 days) of treatment (Figure 2). Case FU049 was female, 33 years of age and diagnosed with a smear grade of 3+ but sputum-smear microscopy grade of scanty 3 at 49 days of follow-up. Case FU031 was male, 51 years of age, and diagnosed with a smear grade of 3+ and had a smear grade of 1+ at 175 days of follow-up. The SNP distances between each genome pair is shown in Figure 2. On average, there were 1.3 (4/3) SNPs accrued in 126 days [(153+49+175)/3]. This implies that, within 3 years, it is possible to accrue approximately 11 SNPs, all things being equal. Consequently, our analysis and inferences were based on a 10 SNP cutoff.

**Figure 3:**
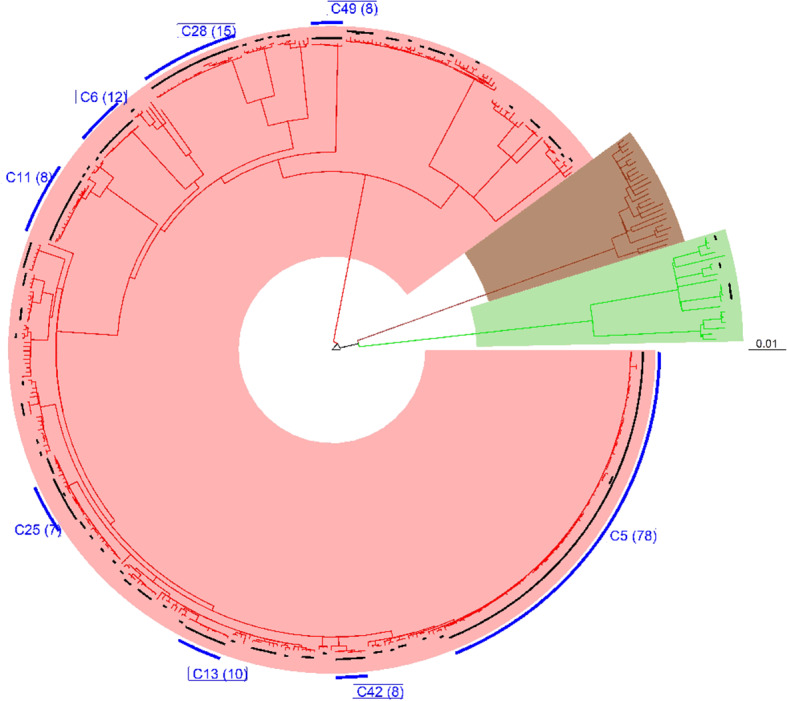
Phylogenetic reconstruction of 452 *M. tuberculosis* complex isolates showing clustering at a threshold of 10 SNPs. The tree was built with an alignment file containing 11,041 variable positions and rooted on *M. canettii*. Black bars plotted on the tips of the branches indicate the clustered cases at the defined threshold of 10 SNPs. Blue bars represent large clusters (cluster size >5) with the number of clustered cases indicated in brackets. The three major branches constitute the three main MTBC lineages found in Ghana and color coded as red for lineage 4, brown for lineage 5, and green for lineage 6.

**Figure 4:**
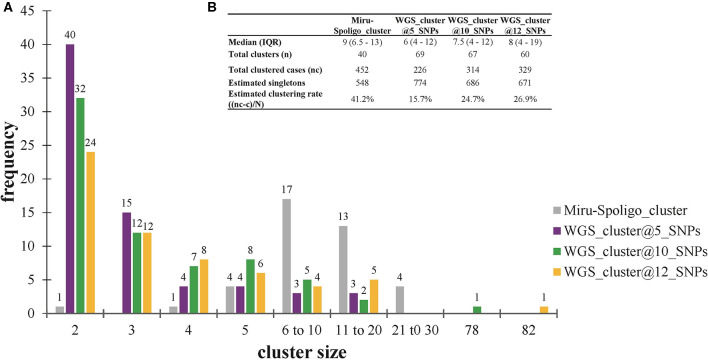
Clustering analysis stratified by cluster definition. **(A)** Frequency of clusters per cluster definition. **(B)** Estimated clustering rate per cluster definition. The estimated population size (*N* = 1000) used for estimating the clustering rate are explained in *Materials and methods* (see Supplementary Data). Singletons were calculated from the estimated population size of 1,000 individuals. IQR, interquartile range.

All 452 genomes were broadly grouped into the three main phylogenetic lineages found in Ghana (lineages 4, 5, and 6) (Figure 3, Supplementary Figures 1–3). The traditional genotype clusters were found to form close to distinct monophyletic clades upon reconstructing the phylogenetic tree using WGS data (Supplementary Figure 1). Some monophyletic clades, however, contained more than one large traditional genotype cluster. Whereas, no cluster of L5 was observed (as per genetic distances), we identified three small clusters of L6 and several clusters for L4. We identified 67 clusters with a median cluster size of 7.5 genomes (range, 4 to 12) and total number of clustered genomes being 314 (Figures 4A,B). Eight large clusters were observed with the largest cluster consisting of 78 genomes (Figure 3, Figure 4A). The estimated clustering rate (recent transmission rate) was 24.7% (Figure 4B). In addition to the SNP threshold at 10, we explored also SNP thresholds at 5 and 12 (Supplementary Figures 2, 3).

### Recent Transmission Hotspots and Characteristics of Large Clusters

**Figure 5:**
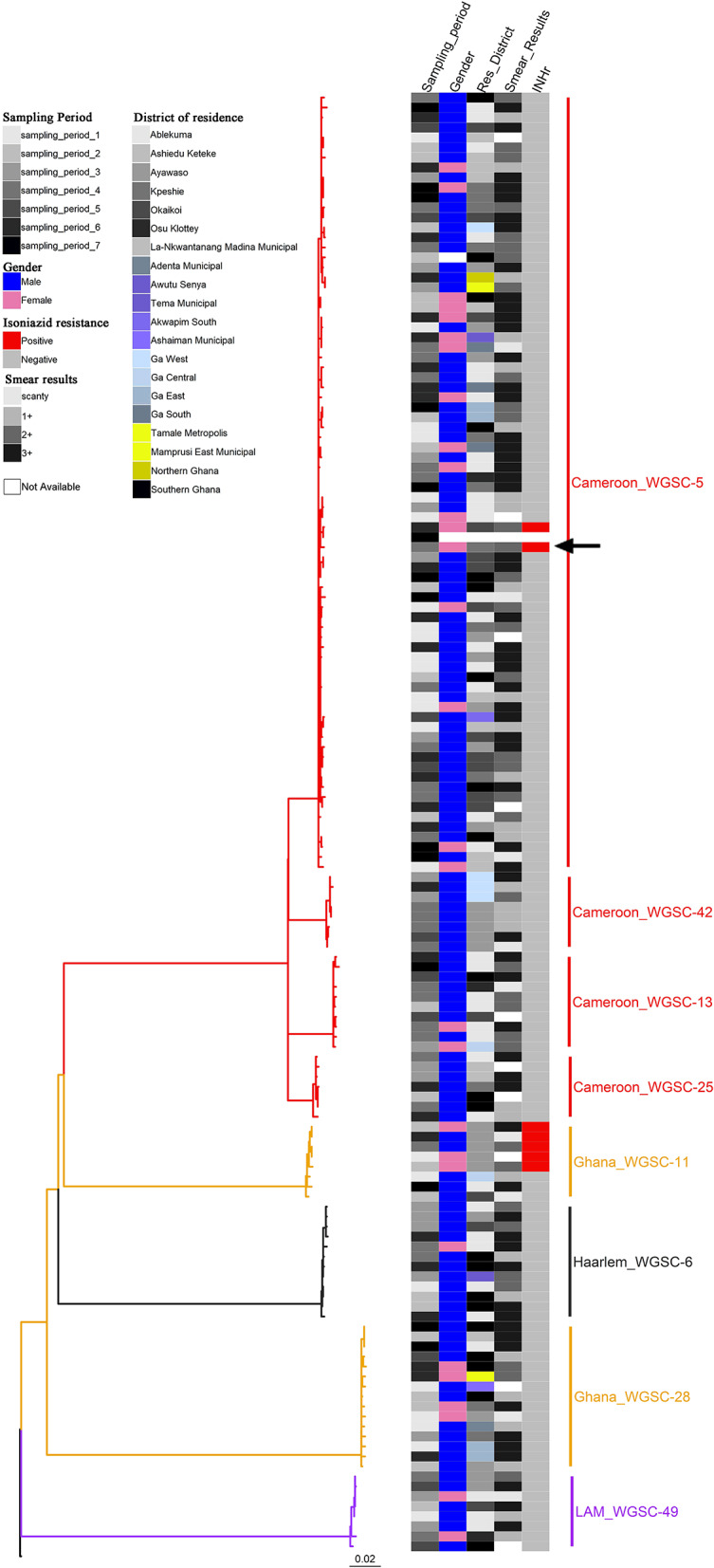
Phylogenetic reconstruction of 146 *M. tuberculosis* complex isolates rooted on *M. canettii* showing characteristics of the eight identified large clusters as defined by a threshold of 10 SNPs. The heat map shows some characteristics of the clustered cases including sampling period (column 1), gender (column 2), residential district (column 3), smear results (column 4), and drug resistance status to isoniazid (column 5). There was only one rifampicin resistant isolate (black arrow). The color codes are defined in the key. All cases belong to lineage 4.

**Figure 6:**
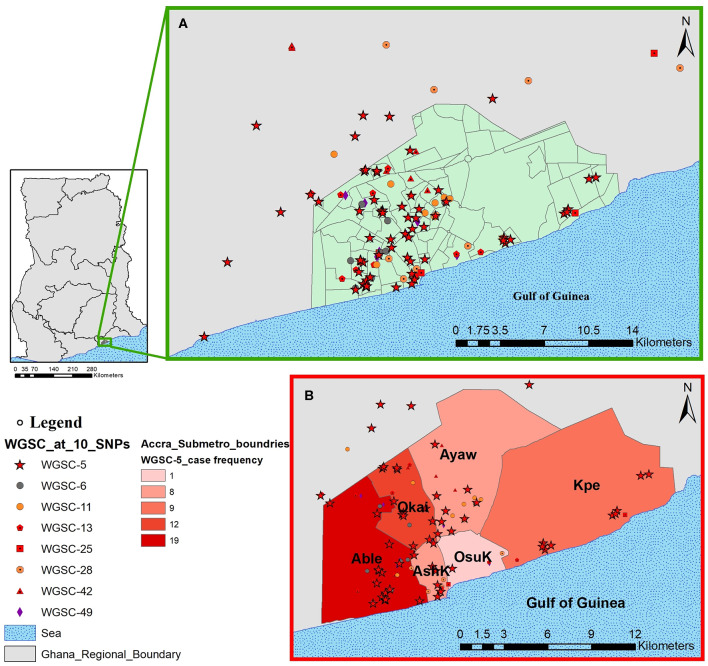
Spatial distribution of pulmonary tuberculosis cases belonging to large clusters in and around Accra metropolis. The geographical distribution of cases from all eight large clusters is shown in **(A)** and the relative distribution of cases from the largest cluster highlighted in **(B)** stratified by Accra metropolis sub-district. Abbreviations: Able, Ablekuma sub-district; Okai, Okaikoi sub-district; AshK, AShiedu Keteke sub-district; OsuK, Osu Klottey sub-district; Ayaw, Ayawaso sub-district; Kpe, Kpeshie sub-district.

A total of 146 genomes constituting eight large clusters (defined as cluster size > 5 in *Materials and methods*) (Table 1 and Figures 2, 5) were observed from the clustering analysis with 10 SNP threshold. The smallest of these large clusters had a cluster size of seven genomes (whole genome sequence cluster 25; WGSC-25), whereas the largest had 78 genomes (WGSC-5), which formed a quarter of all clustered cases (78/314, 25%). All the large clusters belonged to lineage 4 with Cameroon sub-lineage predominating (WGSC-5, WGSC-13, WGSC-25, and WGSC-42) followed by the Ghana sub-lineage (WGSC-11 and WGSC-28). The two remaining large clusters belonged to the Haarlem (WGSC-6) and LAM (WGSC-49) sub-lineages. Not more than 138 variable SNPs were observed within these large clusters. The median pairwise SNP distance within these large clusters was seven SNPs. Apart from 7 isolates, all remaining 139/146 isolates were sensitive to both isoniazid and rifampicin. Interestingly, 5/7 INH-resistant isolates belonged to the same cluster (WGSC-11) and were from individuals residing in the same sub-district (Ayawaso) (Figures 5, 6). Only two isolates belonging to WGSC-5 were resistant to INH. The ratio of male to female cases infected with the clustered genomes was confirmed to be significantly higher (3:1, 231/81) compared to the general population (2:1) and twice as much among large clusters (4:1, 115/29) (*p* < 0.05). Two participants had no record of gender. Two large clusters (WGSC-25 and WGSC-42) were made up of only males.

**Table 1:** Characteristics and risk factor analysis of large genomic clusters resulting from a threshold of 10 SNPs.

**Number**	**WGS cluster code**	**Number of cases in cluster**	**Number of variable fixed SNPs**	**Median pairwise SNP (IQR)**	**Lineage (sub-lineage^**a**^)**	**Lineage classification by Stucki/Coll**	**Any drug resistance^**b**^**	**Gender, male:female**	**Median age (IQR)**
1	WGSC-5	78	138	7 (6–9)	L4 (Cameroon)	L4.6.2/L4.6.2.2	2	59:17	34 (24–43)
2	WGSC-28	15	39	7 (5–7)	L4 (Ghana)	L4.10/L4.8	ND	11:4	39 (32–51)
3	WGSC-6	12	18	5 (3–6)	L4 (Haarlem)	NA/L4.6	ND	11:1	38 (28–48)
4	WGSC-13	10	23	5 (5–6)	L4 (Cameroon)	L4.6.2/L4.6.2.2	ND	8:2	42.5 (32–49)
5	WGSC-11	8	22	8 (6.5–8.5)	L4 (Ghana)	NA/L4.6.2	5	5:3	32.5 (28–41.5)
6	WGSC-42	8	6	3 (1–3.5)	L4 (Cameroon)	L4.6.2/L4.6.2.2	ND	8:0	25.5 (22.5–28.5)
7	WGSC-49	8	13	4 (1–7.5)	L4 (LAM)	L4.3/L4.3.1	ND	6:2	42 (32–54)
8	WGSC-25	7	16	4 (3–9)	L4 (Cameroon)	L4.6.2/L4.6.2.2	ND	7:0	39 (28–50)

WGS, Whole genome sequencing; L4, lineage 4; ND, none determined; IQR, interquartile range.

a Sub-lineage defined using spoligotyping.

b *Number of participants carrying strains with drug resistance to either isoniazid or rifampicin*.

All cases belonging to large clusters spanned the entire 3.5-year sampling period and were distributed among 20 districts/sub-districts but generally clustered within Accra metropolis (Figures 5, 6), which is made up of six sub-districts. Most of the large clusters exhibited a geographically clustered distribution even though not exclusive. For example, whereas hotspot for WGSC-11 and WGSC-42 was the Ayawaso sub-district, WGSC-13 and WGSC-5 were found mostly in the Ablekuma sub-district, the main identified hotspot of recent transmission (Supplementary Figure 4 and Supplementary Table 2).

### Socio-Demographic Characteristics of Individuals Infected With a Strain From the Largest Cluster (WGSC-5)

This largest transmitting cluster made up of 78 cases exhibited an interesting geographical distribution. Except for two cases from Northern Ghana, all 76/78 cases in this cluster were from Southern Ghana of which 19 were found in Ablekuma (Figures 5, 6B and Supplementary Figure 4). The two cases from Northern Ghana shared no SNP difference between them. One case had no record of residential location. There were 59 males and 17 females with a median age of 34 (IQR, 24–43). Two participants had no record of gender. A greater proportion (77.8%, 42/54) of individuals responded living in compound houses at city suburb (66.1%, 37/56) with an average monthly income of not more than 300 Ghanaian cedis (92.8%, 52/56) or 60 USD in its equivalence. The median number of individuals living in a giving household was 12 (IQR, 5–20). On average, there were more unskilled laborers (60.7%, 34/56) than skilled laborers (16.1%, 9/56) with the remaining 23.2% (13/56) being unemployed including students.

## Discussion

In this study, our main goal was to use a WGS approach to resolve large traditional genotype clusters (MIRU/Spoligo defined clusters) and explore some epidemiological characteristics including spatial distribution of confirmed large clusters. Major findings from our analysis indicate that (1) estimated recent TB transmission rate using WGS at a SNP threshold of 10 remains high at 24.7%, and (2) there is wide spread of a clone of the Cameroon sub-lineage of lineage 4 with an ongoing transmission at hotspots mostly found within the Ablekuma sub-district of the Accra metropolis.

WGS was first used in 2011 to delineate two unrelated transmission events among a cohort of drug users with identical MIRU-VNTR profiles from Vancouver and ever since it has been used in some large studies to understand TB transmission dynamics (34–36). Despite the continuous progress and decreasing costs of WGS-based typing, there are some important pertaining challenges such as the lack of standardization of WGS analysis pipelines and genomic distances (SNP distance) for defining clusters (37). A first step in analyzing WGS data for transmission studies is usually to define SNP threshold to identify cluster, and the assumption is that, isolates from cases separated by SNPs less than or equal to the specified threshold are epidemiologically linked (38). The mutation rate established from our within-host microevolution analysis using the main lineage/sub-lineage population, suggested a cut-off at 11 SNPs will be adequate to define a cluster. Consequently, we chose a SNP threshold of 10 for our analysis. This chosen threshold is ideal as other similarly high TB transmission settings like Malawi, have used the same threshold to infer recent transmission (27). Though our genome coverage cut-off was 15x, we had an overall mean genome coverage of 77x (± 27x) for all 452 genomes. Our cut-off is similar to that used in comparable studies which based their analysis on genome coverage cut-off of 10x, 15x, or 20x (27, 39–42).

We previously estimated the recent transmission index to be 41.2%, using MIRU/Spoligo which is higher than the current estimate of 24.7% using WGS analysis. This reduced rate was anticipated as the discriminatory power of WGS analysis is higher (43). Nevertheless, the 24.7% estimated recent transmission rate is still high comparable to 30% from a similarly high transmission setting like Malawi (27, 44) and predicts the occurrence of undetected recent transmission of large clusters. With the exception of three clusters of lineage 6, all the remaining 64 clusters were lineage 4 and no cluster from lineage 5. This finding confirms our previous report of reduced recent transmission of MAF lineages (L5 and L6) compared to MTBs and has stressed the need for further studies to investigate the continuous prevalence of MAF in West Africa. The observation of nearly distinct monophyletic clades from the reconstructed phylogenetic tree implies that traditional genotyping may still be useful as initial screening tools to help reduce the huge cost of WGS of all isolates especially in large-size population-based studies.

Within our study population, we did not identify any cluster consisting of multidrug-resistant strains, confirming our previous report of the unlikeliness of a drug-resistant TB strain to be involved in a recent transmission event (10). This observation may be due to the low proportion (2–4%) of MDR among MTBC isolates in Ghana (10, 11) or probably due to the reduced fitness cost associated to resistance conferring mutations (45, 46). Moreover, only 7/146 cases belonging to large genomic clusters were resistant to INH. Interestingly, 5/7 INH-resistant isolates belonged to the same Ghana sub-lineage cluster (WGSC-11). The Ghana sub-lineage has previously been associated with drug resistance (5, 11). Though the size of the cluster is not very large (cluster size of 8), this is nonetheless worrying since recent transmission of such drug-resistant clone may pose a great challenge to TB control in the sub-region. Until recently, drug-resistant clones were thought to be less fit and less likely to transmit from person to person; however, recent studies have documented evidence of transmission even though not involving large clusters (42, 47, 48). There is therefore the need to identify and control such difficult-to-treat drug-resistant clones to stop their spread.

Our population-based study included two distant regions in Ghana; the Northern region (in Northern Ghana) and the Greater Accra region (in Southern Ghana). Except for three cases from Northern Ghana (Supplementary Figure 4 and Supplementary Table 2), all 146 cases belonging to large genomic clusters were found in Southern Ghana. Two of the only three large genomic clustered cases from Northern Ghana were found within the largest cluster (WGSC-5, Figure 5). These cases were, however, very closely related, sharing the same most recent common ancestral node and in fact no SNP difference between them, suggesting direct person-to-person transmission. A careful examination of their demographic data also showed that, indeed, these two individuals have the same family name and most probably comes from the same family. We show that the clustering of TB cases in Ablekuma observed in our previous study (5) was most probably due to recent TB transmission. This is not surprising as Ablekuma is the most densely populated of the six sub-districts of Accra metropolis (49). Our analysis suggests that there may be super-spreaders in Ablekuma and probably Okaikoi, which recorded the second highest numbers (19 and 12, respectively; Figure 6 and Supplementary Figure 4) that belonged to the largest cluster (WGSC-5). Majority of the individuals in this high transmitting cluster were found to inhabit the city suburbs in densely populated compound houses. Their low-income status combined with over-crowding may be driving factors for the ongoing transmission in this hotspot. A high smear grade of over 70% of cases being at least 2+ signifies that these individuals are likely to have been actively transmitting the pathogen prior to diagnosis in their homes and neighboring communities, indicating that other individuals may have been infected.

The goal of universal screening is what most TB control programs are geared toward especially detecting MDR cases. Our study has identified hotspots not only for recent TB transmission of drug-sensitive strains but also spread of INH-resistant strain. We encourage more similar studies as it can identify geographical zones of highest need to support the national TB control program (NTP) with a targeted and guided approach to controlling TB. Case search approaches targeted at high-risk areas may be more effective in TB control (50). We have shown that application of WGS in a molepi study has aided the recognition of specific *M. tuberculosis* strains (e.g., cluster WGSC-11 associated with drug resistance), which can be predictive of INH drug-resistant TB in the Ghanaian contexts that could and can help provide indications of the TB case source similar to TB strains elsewhere (51). Also, we have been able to identify hotspots of recent TB transmission within the Accra Metropolis; hence, we recommend an urgent action to curtail the continual spread of the pathogen.

## Data Availability Statement

The raw sequence data are available under the BioProject ID: PRJNA616081 with the various accession numbers specified in Supplementary Table 1.

## Ethics Statement

The studies involving human participants were reviewed and approved by Scientific and Technical Committee and the Institutional Review Board of Noguchi Memorial Institute for Medical Research, University of Ghana (FWA00001824). Written informed consent to participate in this study was provided by the participants' legal guardian/next of kin.

## Author Contributions

DY-M designed the study, provided supervision and support, provided intellectual input, and wrote the manuscript. PA performed most of the laboratory procedures, collated the epidemiological and laboratory data, did all statistical and cluster analysis on the data, and wrote the manuscript. SO-W performed some laboratory procedures and provided all associated data and provided useful comments to writing the manuscript. NB and AF supported the enrollment and collection of clinical and demographic data from the health facilities. EB, MR, and DP performed some laboratory procedures and provided all associated data and helped with preliminary analysis. IO performed some laboratory procedures and provided all associated data, performed some analysis, and provided useful comments to writing the manuscript. DB, CL, and SB provided supervision, support, and intellectual input, and critically reviewed the manuscript. AA-P contributed to the study design, performed some laboratory procedures, and provided all associated data and provided useful comments to writing the manuscript. KK provided supervision and support, intellectual input, and useful comments to writing the manuscript. SG designed the study, provided supervision and support, provided intellectual input, and critically reviewed the manuscript. All coauthors reviewed and approved the final manuscript before submission.

## Conflict of Interest

The authors declare that the research was conducted in the absence of any commercial or financial relationships that could be construed as a potential conflict of interest.
