# Supplementary material for: Whole Genome Sequencing and Spatial Analysis Identifies Recent Tuberculosis Transmission Hotspots in Ghana
Source: Front Med (Lausanne). 2020 May 19;7:161. doi: 10.3389/fmed.2020.00161 (PMC7248928; doi:10.3389/fmed.2020.00161)
Supplement: Supplementary file 1 [file Data_Sheet_1.docx]

**Supplementary data**

**Recent TB transmission rate and population size estimation**

The recent TB transmission rate was estimated using the n – 1 formula described by Glynn et al., 1999; $\frac{(nc-c)}{N}$ where nc is the total number of clustered cases, c is the number of clusters, and N is the total number of cases in the sample [1].

Our sample set for this current study constituted a biased collection of only traditional genotype clustered cases. In order to estimate a recent transmission rate from the WGS clustering analysis, we first estimated the number of single cases from the previous population as follows;

If the previous population size of 2,309 isolates resulted in an estimated traditional genotypic recent transmission rate of 41.2% from 1,227 clustered isolates of 276 clusters (thus, ((1227-276)/2309) = 41.2%), then a selected traditional genotypic clustered cases of 452 strains from 40 clustered cases will have been drawn from a population size of N given by;

$$\frac{(nc-c)}{N}=41.2\%$$

But nc = 452 and c = 40, hence

$$\frac{(452-40)}{N}=\frac{41.2}{100}$$

Computing gives N = 1000 isolates.

**Reference**

1. Glynn JR, Vynnycky E, Fine PE. Influence of sampling on estimates of clustering and recent transmission of *Mycobacterium tuberculosis* derived from DNA fingerprinting techniques. Am J Epidemiol. 1999;149(4):366-71.


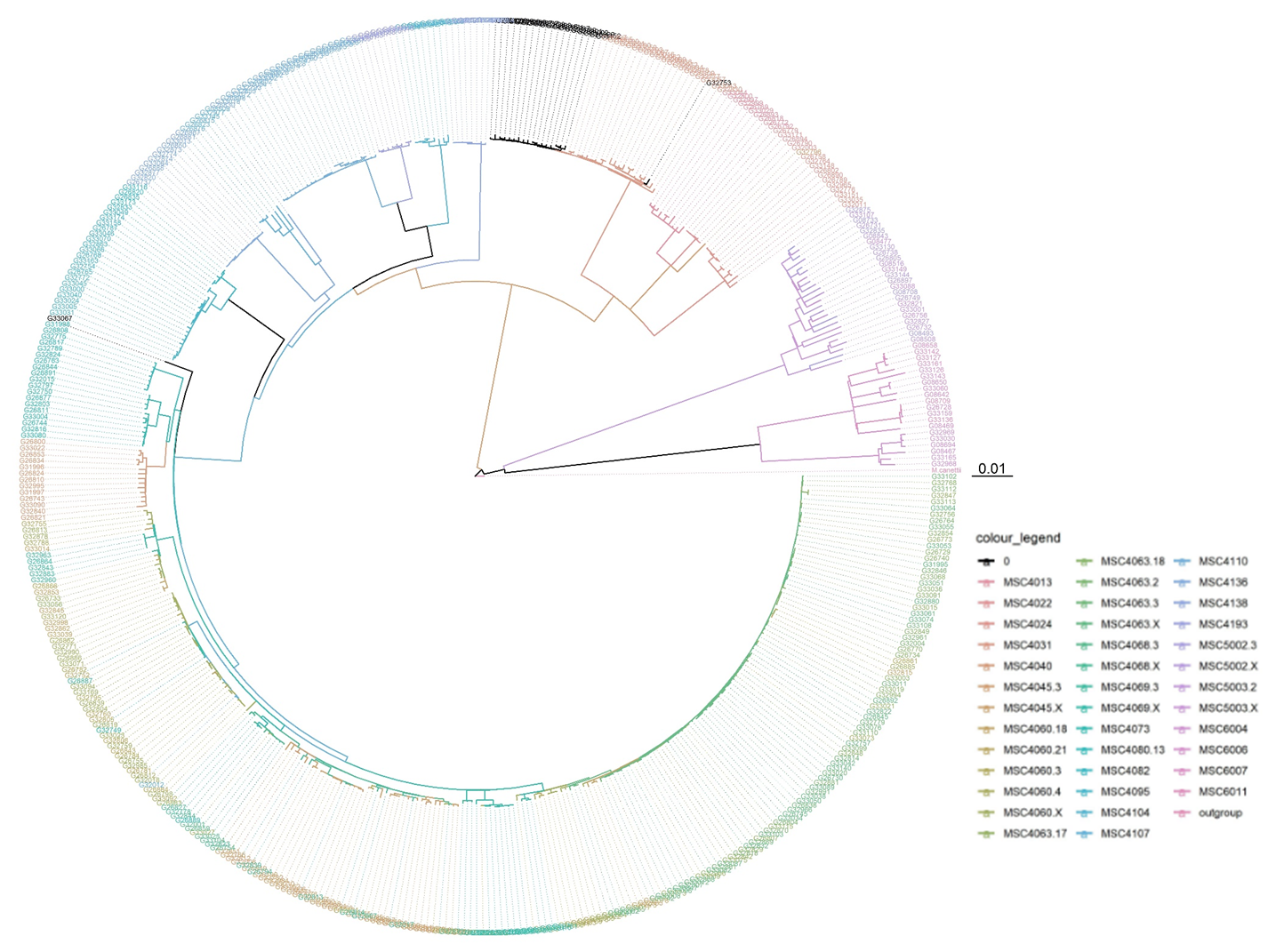


**Supplementary figure 1.** Clustering and phylogenomic relationship of 452 *M. tuberculosis* complex isolates. The color codes represent clusters previously defined by combined resolution of MIRU-VNTR typing and spoligotyping, here referred to as traditional genotype clusters. Near distinct monophylectic clades represent the various large traditional genotype clusters. The tree was rooted with *M. canettii*.


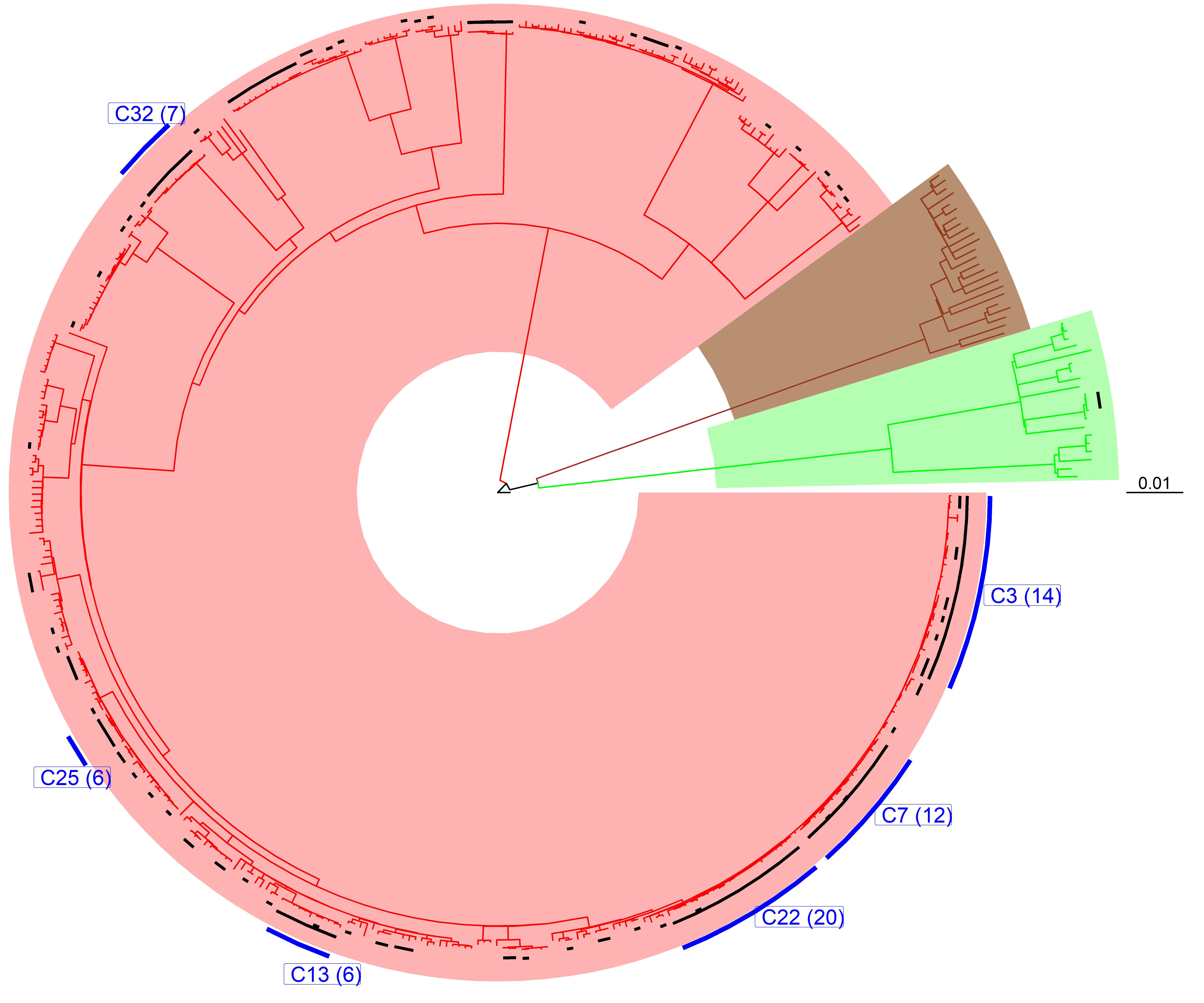


**Supplementary figure 2.** Phylogenetic reconstruction of 452 *M. tuberculosis* complex isolates showing clustering at a threshold of 5-SNPs. The tree was built with an alignment file containing 11,041 variable positions. Black bars plotted on the tips of the branches indicate the clustered cases at the defined threshold of 5-SNPs. Blue bars represent large clusters (cluster size >5) with the number of clustered cases indicated in brackets. The three major branches constitute the three main MTBC lineages found in Ghana and color coded as red for lineage 4, brown for lineage 5 and green for lineage 6. The tree was rooted with *M. canettii*. Using a SNP threshold of 5, we identified 69 clusters with a median cluster size of 6 (range 4 to 12) and total clustered cases of 226 individuals. Six large clusters were observed. All the large clusters were supported with a bootstrap value of 100.


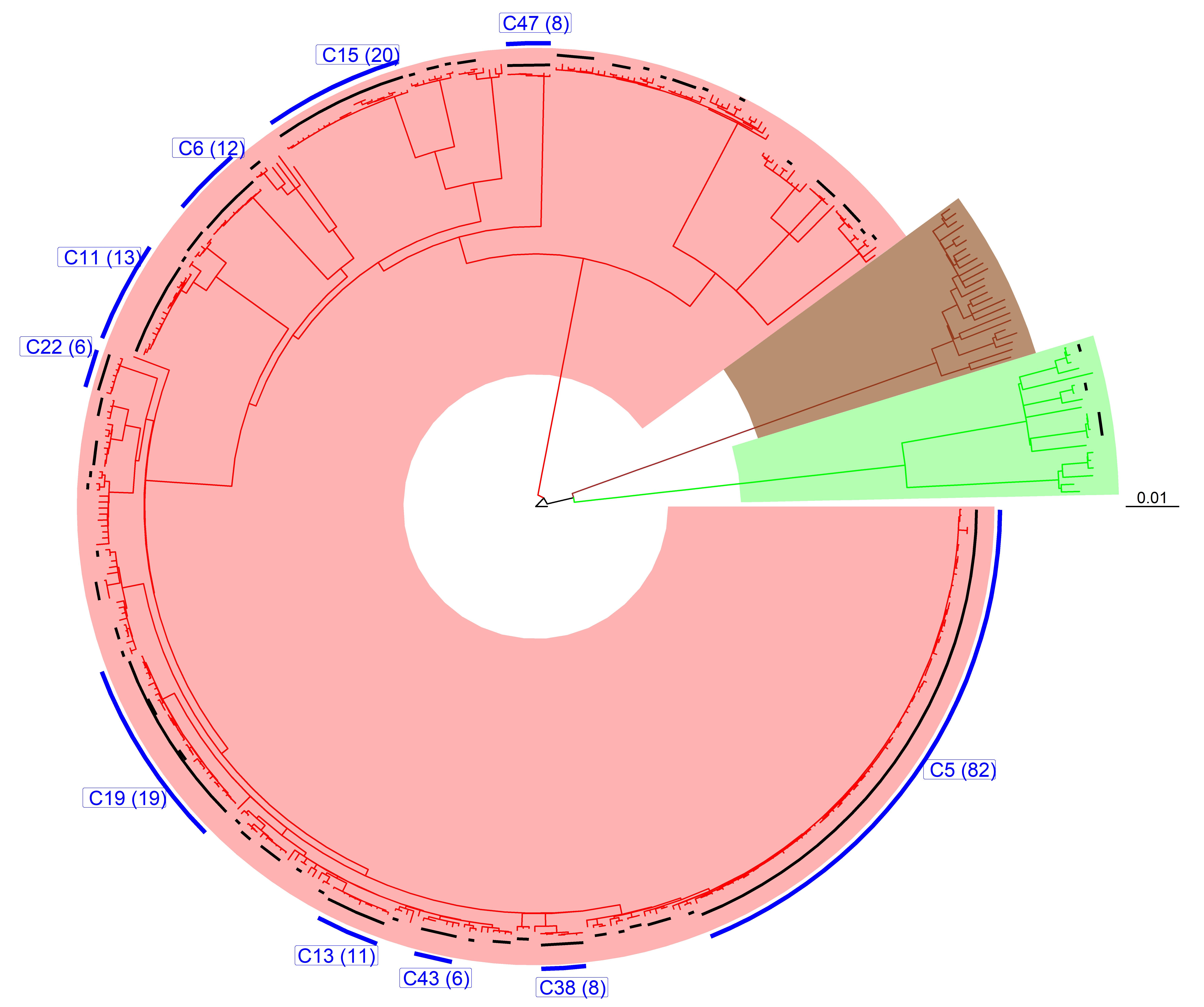


**Supplementary figure 3.** Phylogenetic reconstruction of 452 *M. tuberculosis* complex isolates showing clustering at a threshold of 12-SNPs. The tree was built with an alignment file containing 11,041 variable positions. Black bars plotted on the tips of the branches indicate the clustered cases at the defined threshold of 12-SNPs. Blue bars represent large clusters (cluster size >5) with the number of clustered cases indicated in brackets. The three major branches constitute the three main MTBC lineages found in Ghana and color coded as red for lineage 4, brown for lineage 5 and green for lineage 6. The tree was rooted with *M. canettii*. Using a SNP threshold of 12, we identified 60 clusters with a median cluster size of 6 (range 4 to 12) and total clustered cases of 329 individuals. Ten large clusters were observed with the largest consisting of 82 genomes. All the large clusters were supported with a bootstrap value of 100.

**Supplementary figure 4**. Frequency and relative geographical distribution of cases in all 8 large clusters identified by a threshold of 10-SNPs.

**Supplementary table 1.** Availability of raw sequence reads

| **Genome number** | **Isolate ID** | **Coverage** | **Standard deviation** | **Isolation Institute** | **Sequencing Institute** | **Provider** | **Accession number** | **Country of origin** |
| --- | --- | --- | --- | --- | --- | --- | --- | --- |
| G08467 | 1108 | 69.33 | 37.24 | NMIMR | WTSI | DYM | ERR1082114 | Ghana |
| G08469 | 2287 | 164.96 | 41.77 | NMIMR | WTSI | DYM | ERR1082116 | Ghana |
| G08477 | 3285 | 141.32 | 25.18 | NMIMR | WTSI | DYM | ERR1082126 | Ghana |
| G08493 | 2464 | 208.89 | 31.48 | NMIMR | WTSI | DYM | ERR1082142 | Ghana |
| G08508 | 2667 | 124.55 | 19.6 | NMIMR | WTSI | DYM | ERR1203066 | Ghana |
| G08509 | 2463 | 136.26 | 29.27 | NMIMR | WTSI | DYM | ERR1203067 | Ghana |
| G08513 | 2584 | 65.27 | 13.57 | NMIMR | WTSI | DYM | ERR1203071 | Ghana |
| G08516 | 2890 | 125.48 | 20.35 | NMIMR | WTSI | DYM | ERR1203074 | Ghana |
| G08642 | 1200 | 76.06 | 14.52 | NMIMR | WTSI | DYM | ERR502522 | Ghana |
| G08650 | 1434 | 92.85 | 17.12 | NMIMR | WTSI | DYM | ERR502533 | Ghana |
| G08658 | 2151 | 72.98 | 17.59 | NMIMR | WTSI | DYM | ERR1215471 | Ghana |
| G08694 | 2016 | 87.71 | 18.04 | NMIMR | WTSI | DYM | ERR751294 | Ghana |
| G08708 | 2010 | 77.59 | 13.77 | NMIMR | WTSI | DYM | ERR751308 | Ghana |
| G08709 | 2150 | 87.51 | 16.4 | NMIMR | WTSI | DYM | ERR751309 | Ghana |
| G08733 | 1166 | 76.14 | 13.88 | NMIMR | WTSI | DYM | ERR751337 | Ghana |
| G08742 | 1030 | 101.09 | 18.76 | NMIMR | WTSI | DYM | ERR751346 | Ghana |
| G08843 | 2556 | 27.27 | 7.62 | NMIMR | WTSI | DYM | ERR1215468 | Ghana |
| G26728 | 2162 | 56.97 | 11.03 | NMIMR | STPH | DYM | SRR11444238 | Ghana |
| G26729 | 2457 | 56.33 | 11.05 | NMIMR | STPH | DYM | SRR11444198 | Ghana |
| G26730 | 1402 | 60.55 | 12.41 | NMIMR | STPH | DYM | SRR11444360 | Ghana |
| G26731 | 3363 | 55.19 | 11.16 | NMIMR | STPH | DYM | SRR11444083 | Ghana |
| G26732 | 1052 | 31.58 | 7.88 | NMIMR | STPH | DYM | SRR11444290 | Ghana |
| G26733 | 4042 | 70.7 | 22.99 | NMIMR | STPH | DYM | SRR11443998 | Ghana |
| G26734 | 1270 | 67.04 | 12.25 | NMIMR | STPH | DYM | SRR11444013 | Ghana |
| G26735 | 4003 | 74.11 | 13.42 | NMIMR | STPH | DYM | SRR11444007 | Ghana |
| G26737 | 2399 | 78.2 | 12.65 | NMIMR | STPH | DYM | SRR11444212 | Ghana |
| G26738 | 3827 | 47.69 | 9.74 | NMIMR | STPH | DYM | SRR11444026 | Ghana |
| G26739 | 4409 | 76.56 | 14.2 | NMIMR | STPH | DYM | SRR11444379 | Ghana |
| G26740 | 3753 | 83.85 | 13.16 | NMIMR | STPH | DYM | SRR11444038 | Ghana |
| G26743 | 1238 | 65.7 | 11.25 | NMIMR | STPH | DYM | SRR11444079 | Ghana |
| G26744 | 2444 | 73.33 | 12.2 | NMIMR | STPH | DYM | SRR11444200 | Ghana |
| G26745 | 4197 | 71.29 | 12.84 | NMIMR | STPH | DYM | SRR11444412 | Ghana |
| G26747 | 2467 | 74.94 | 12.38 | NMIMR | STPH | DYM | SRR11444196 | Ghana |
| G26749 | 2219 | 62.94 | 10.52 | NMIMR | STPH | DYM | SRR11444232 | Ghana |
| G26750 | 2731 | 68.27 | 10.3 | NMIMR | STPH | DYM | SRR11444152 | Ghana |
| G26754 | 4173 | 79.92 | 12.45 | NMIMR | STPH | DYM | SRR11443983 | Ghana |
| G26755 | 1020 | 78.1 | 12.99 | NMIMR | STPH | DYM | SRR11443980 | Ghana |
| G26756 | 1313 | 79.79 | 13.84 | NMIMR | STPH | DYM | SRR11444372 | Ghana |
| G26758 | 4238 | 82.39 | 11.69 | NMIMR | STPH | DYM | SRR11444401 | Ghana |
| G26763 | 1760 | 70.02 | 12.32 | NMIMR | STPH | DYM | SRR11444295 | Ghana |
| G26764 | 1376 | 78.23 | 13.88 | NMIMR | STPH | DYM | SRR11444365 | Ghana |
| G26765 | 3764 | 61.93 | 9.7 | NMIMR | STPH | DYM | SRR11444036 | Ghana |
| G26768 | 3742 | 82.47 | 10.95 | NMIMR | STPH | DYM | SRR11444040 | Ghana |
| G26769 | 1836 | 68.17 | 10.25 | NMIMR | STPH | DYM | SRR11444285 | Ghana |
| G26770 | 4009 | 69.26 | 11.73 | NMIMR | STPH | DYM | SRR11444004 | Ghana |
| G26771 | 1570 | 68.4 | 12.03 | NMIMR | STPH | DYM | SRR11444340 | Ghana |
| G26772 | 3616 | 66.17 | 10.68 | NMIMR | STPH | DYM | SRR11444063 | Ghana |
| G26773 | 1284 | 62.41 | 11.21 | NMIMR | STPH | DYM | SRR11444392 | Ghana |
| G26779 | 1785 | 66.43 | 13.22 | NMIMR | STPH | DYM | SRR11444293 | Ghana |
| G26781 | 1758 | 68.32 | 11.67 | NMIMR | STPH | DYM | SRR11444296 | Ghana |
| G26782 | 4349 | 69.35 | 12.05 | NMIMR | STPH | DYM | SRR11444385 | Ghana |
| G26783 | 2753 | 72.41 | 10.8 | NMIMR | STPH | DYM | SRR11444147 | Ghana |
| G26784 | 1346 | 56.27 | 10.28 | NMIMR | STPH | DYM | SRR11444368 | Ghana |
| G26787 | 1636 | 65.74 | 11.33 | NMIMR | STPH | DYM | SRR11444331 | Ghana |
| G26788 | 1065 | 61.33 | 10.71 | NMIMR | STPH | DYM | SRR11444279 | Ghana |
| G26789 | 1156 | 65.82 | 11.56 | NMIMR | STPH | DYM | SRR11444146 | Ghana |
| G26790 | 2115 | 67.43 | 10.66 | NMIMR | STPH | DYM | SRR11444242 | Ghana |
| G26791 | 3026 | 77.6 | 11.64 | NMIMR | STPH | DYM | SRR11444118 | Ghana |
| G26792 | 1700 | 63.55 | 10.18 | NMIMR | STPH | DYM | SRR11444310 | Ghana |
| G26793 | 2321 | 66.32 | 10.78 | NMIMR | STPH | DYM | SRR11444217 | Ghana |
| G26794 | 1444 | 53.88 | 12.73 | NMIMR | STPH | DYM | SRR11444353 | Ghana |
| G26796 | 4162 | 69.65 | 12.51 | NMIMR | STPH | DYM | SRR11443986 | Ghana |
| G26797 | 2070 | 67.67 | 10.24 | NMIMR | STPH | DYM | SRR11444249 | Ghana |
| G26798 | 3295 | 65.67 | 11.08 | NMIMR | STPH | DYM | SRR11444093 | Ghana |
| G26799 | 2886 | 55.3 | 11.12 | NMIMR | STPH | DYM | SRR11444134 | Ghana |
| G26800 | 3098 | 71.8 | 11.22 | NMIMR | STPH | DYM | SRR11444111 | Ghana |
| G26801 | 1095 | 65.97 | 10.45 | NMIMR | STPH | DYM | SRR11444224 | Ghana |
| G26802 | 1964 | 75.04 | 11.41 | NMIMR | STPH | DYM | SRR11444262 | Ghana |
| G26803 | 4002 | 69.58 | 10.44 | NMIMR | STPH | DYM | SRR11444008 | Ghana |
| G26804 | 1074 | 61.65 | 14.45 | NMIMR | STPH | DYM | SRR11444246 | Ghana |
| G26805 | 2426 | 69.52 | 12.03 | NMIMR | STPH | DYM | SRR11444206 | Ghana |
| G26806 | 2716 | 54.23 | 10.77 | NMIMR | STPH | DYM | SRR11444155 | Ghana |
| G26807 | 1017 | 74.61 | 12.64 | NMIMR | STPH | DYM | SRR11444202 | Ghana |
| G26808 | 1910 | 70.9 | 11.57 | NMIMR | STPH | DYM | SRR11444271 | Ghana |
| G26809 | 3208 | 66.51 | 11.29 | NMIMR | STPH | DYM | SRR11444101 | Ghana |
| G26810 | 2272 | 71.56 | 11.53 | NMIMR | STPH | DYM | SRR11444223 | Ghana |
| G26811 | 4049 | 38.2 | 7.79 | NMIMR | STPH | DYM | SRR11443997 | Ghana |
| G26812 | 2735 | 69.93 | 11.96 | NMIMR | STPH | DYM | SRR11444150 | Ghana |
| G26813 | 2409 | 62.55 | 11.97 | NMIMR | STPH | DYM | SRR11444208 | Ghana |
| G26814 | 3889 | 64.23 | 12.31 | NMIMR | STPH | DYM | SRR11444017 | Ghana |
| G26815 | 1742 | 66.07 | 11.77 | NMIMR | STPH | DYM | SRR11444299 | Ghana |
| G26816 | 3296 | 72.03 | 11.23 | NMIMR | STPH | DYM | SRR11444092 | Ghana |
| G26817 | 2661 | 72.32 | 11.6 | NMIMR | STPH | DYM | SRR11444165 | Ghana |
| G26818 | 1845 | 63.92 | 12.18 | NMIMR | STPH | DYM | SRR11444283 | Ghana |
| G26819 | 2976 | 72.38 | 13.39 | NMIMR | STPH | DYM | SRR11444125 | Ghana |
| G26820 | 1718 | 69.37 | 12.05 | NMIMR | STPH | DYM | SRR11444303 | Ghana |
| G26821 | 2367 | 67.39 | 13.21 | NMIMR | STPH | DYM | SRR11444216 | Ghana |
| G26822 | 3849 | 67.02 | 11.71 | NMIMR | STPH | DYM | SRR11444023 | Ghana |
| G26823 | 2825 | 65.97 | 11.05 | NMIMR | STPH | DYM | SRR11444140 | Ghana |
| G26824 | 1614 | 67.77 | 10.82 | NMIMR | STPH | DYM | SRR11444334 | Ghana |
| G26826 | 1536 | 65.49 | 10.04 | NMIMR | STPH | DYM | SRR11444344 | Ghana |
| G26827 | 3088 | 68.25 | 12.96 | NMIMR | STPH | DYM | SRR11444114 | Ghana |
| G26828 | 1904 | 112.5 | 19.55 | NMIMR | STPH | DYM | SRR11444272 | Ghana |
| G26829 | 1281 | 34.11 | 7.52 | NMIMR | STPH | DYM | SRR11444403 | Ghana |
| G26830 | 1731 | 94.68 | 18.21 | NMIMR | STPH | DYM | SRR11444300 | Ghana |
| G26833 | 2766 | 106.45 | 18.68 | NMIMR | STPH | DYM | SRR11444145 | Ghana |
| G26834 | 1196 | 94.56 | 17.3 | NMIMR | STPH | DYM | SRR11444124 | Ghana |
| G26835 | 1645 | 102.01 | 18.39 | NMIMR | STPH | DYM | SRR11444328 | Ghana |
| G26836 | 2400 | 95.11 | 15.8 | NMIMR | STPH | DYM | SRR11444211 | Ghana |
| G26837 | 1687 | 34.55 | 7.35 | NMIMR | STPH | DYM | SRR11444317 | Ghana |
| G26838 | 1798 | 76.17 | 16.15 | NMIMR | STPH | DYM | SRR11444289 | Ghana |
| G26839 | 2721 | 85.79 | 15.68 | NMIMR | STPH | DYM | SRR11444154 | Ghana |
| G26841 | 1563 | 134.09 | 20.23 | NMIMR | STPH | DYM | SRR11444341 | Ghana |
| G26842 | 2771 | 90.9 | 16.28 | NMIMR | STPH | DYM | SRR11444144 | Ghana |
| G26843 | 2269 | 103.02 | 19 | NMIMR | STPH | DYM | SRR11444225 | Ghana |
| G26844 | 3746 | 96.09 | 15.75 | NMIMR | STPH | DYM | SRR11444039 | Ghana |
| G26845 | 1430 | 133.87 | 22.67 | NMIMR | STPH | DYM | SRR11444355 | Ghana |
| G26846 | 3706 | 76.87 | 15.72 | NMIMR | STPH | DYM | SRR11444043 | Ghana |
| G26847 | 3669 | 80.07 | 21.83 | NMIMR | STPH | DYM | SRR11444052 | Ghana |
| G26848 | 2441 | 123.89 | 19.42 | NMIMR | STPH | DYM | SRR11444203 | Ghana |
| G26849 | 2055 | 98 | 17.22 | NMIMR | STPH | DYM | SRR11444250 | Ghana |
| G26850 | 2025 | 142.02 | 22.48 | NMIMR | STPH | DYM | SRR11444254 | Ghana |
| G26851 | 1272 | 96.61 | 20.44 | NMIMR | STPH | DYM | SRR11444002 | Ghana |
| G26852 | 1049 | 34.51 | 7.57 | NMIMR | STPH | DYM | SRR11444301 | Ghana |
| G26853 | 3939 | 87.66 | 15.86 | NMIMR | STPH | DYM | SRR11444011 | Ghana |
| G26858 | 1685 | 95.97 | 17.74 | NMIMR | STPH | DYM | SRR11444318 | Ghana |
| G26859 | 3241 | 93.06 | 16.14 | NMIMR | STPH | DYM | SRR11444098 | Ghana |
| G26860 | 2623 | 35.55 | 8.36 | NMIMR | STPH | DYM | SRR11444172 | Ghana |
| G26861 | 1985 | 96.66 | 17.33 | NMIMR | STPH | DYM | SRR11444259 | Ghana |
| G26862 | 3439 | 111.7 | 18.31 | NMIMR | STPH | DYM | SRR11444074 | Ghana |
| G26864 | 4278 | 124.8 | 20.18 | NMIMR | STPH | DYM | SRR11444395 | Ghana |
| G26865 | 2668 | 96.23 | 16.04 | NMIMR | STPH | DYM | SRR11444164 | Ghana |
| G26866 | 3346 | 94.48 | 18.1 | NMIMR | STPH | DYM | SRR11444084 | Ghana |
| G26867 | 4294 | 96.88 | 17.71 | NMIMR | STPH | DYM | SRR11444389 | Ghana |
| G26868 | 2726 | 138.21 | 22.67 | NMIMR | STPH | DYM | SRR11444153 | Ghana |
| G26869 | 2602 | 136.27 | 21.11 | NMIMR | STPH | DYM | SRR11444174 | Ghana |
| G26873 | 1223 | 70.87 | 11.48 | NMIMR | STPH | DYM | SRR11444090 | Ghana |
| G26876 | 1829 | 124.49 | 20.64 | NMIMR | STPH | DYM | SRR11444286 | Ghana |
| G26877 | 2197 | 119.72 | 18.88 | NMIMR | STPH | DYM | SRR11444234 | Ghana |
| G26879 | 2928 | 38.04 | 8.43 | NMIMR | STPH | DYM | SRR11444131 | Ghana |
| G26880 | 4408 | 106.02 | 16.26 | NMIMR | STPH | DYM | SRR11444380 | Ghana |
| G26881 | 1754 | 114.38 | 16.85 | NMIMR | STPH | DYM | SRR11444297 | Ghana |
| G26882 | 1029 | 95.6 | 16.52 | NMIMR | STPH | DYM | SRR11444347 | Ghana |
| G26883 | 1016 | 112.69 | 19.58 | NMIMR | STPH | DYM | SRR11444313 | Ghana |
| G26884 | 1675 | 113.82 | 18.6 | NMIMR | STPH | DYM | SRR11444321 | Ghana |
| G26885 | 3422 | 98.84 | 17.28 | NMIMR | STPH | DYM | SRR11444077 | Ghana |
| G26886 | 3239 | 104.81 | 17.42 | NMIMR | STPH | DYM | SRR11444099 | Ghana |
| G26887 | 2246 | 118.6 | 18.84 | NMIMR | STPH | DYM | SRR11444229 | Ghana |
| G26888 | 3781 | 115.87 | 16.03 | NMIMR | STPH | DYM | SRR11444033 | Ghana |
| G26889 | 3811 | 124.52 | 19.11 | NMIMR | STPH | DYM | SRR11444028 | Ghana |
| G26890 | 1261 | 122.24 | 20.92 | NMIMR | STPH | DYM | SRR11444035 | Ghana |
| G26891 | 2406 | 107.36 | 17.42 | NMIMR | STPH | DYM | SRR11444209 | Ghana |
| G26892 | 3634 | 124.86 | 20.54 | NMIMR | STPH | DYM | SRR11444060 | Ghana |
| G26893 | 2186 | 134.16 | 19.42 | NMIMR | STPH | DYM | SRR11444236 | Ghana |
| G26894 | 2015 | 119.6 | 16.69 | NMIMR | STPH | DYM | SRR11444256 | Ghana |
| G26897 | 2028 | 77.28 | 12.2 | NMIMR | STPH | DYM | SRR11444253 | Ghana |
| G26898 | 3025 | 120.6 | 18.7 | NMIMR | STPH | DYM | SRR11444119 | Ghana |
| G31995 | 4221 | 43.74 | 9.64 | NMIMR | STPH | DYM | SRR11444405 | Ghana |
| G31996 | 2539 | 85.44 | 18.94 | NMIMR | STPH | DYM | SRR11444182 | Ghana |
| G31997 | 1973 | 85.89 | 17.22 | NMIMR | STPH | DYM | SRR11444261 | Ghana |
| G31998 | 2306 | 116.46 | 17.44 | NMIMR | STPH | DYM | SRR11444219 | Ghana |
| G31999 | 4219 | 34.55 | 8.06 | NMIMR | STPH | DYM | SRR11444406 | Ghana |
| G32000 | 3399 | 120.91 | 17.9 | NMIMR | STPH | DYM | SRR11444081 | Ghana |
| G32001 | 2625 | 37.51 | 8.05 | NMIMR | STPH | DYM | SRR11444170 | Ghana |
| G32002 | 1797 | 35.24 | 8.21 | NMIMR | STPH | DYM | SRR11444291 | Ghana |
| G32003 | 1286 | 136.04 | 20.03 | NMIMR | STPH | DYM | SRR11444381 | Ghana |
| G32004 | 2529 | 93.82 | 18.53 | NMIMR | STPH | DYM | SRR11444184 | Ghana |
| G32007 | 1666 | 126.14 | 16.49 | NMIMR | STPH | DYM | SRR11444324 | Ghana |
| G32008 | 1870 | 129.03 | 24.04 | NMIMR | STPH | DYM | SRR11444280 | Ghana |
| G32010 | 3623 | 38.1 | 7.98 | NMIMR | STPH | DYM | SRR11444062 | Ghana |
| G32011 | 3150 | 128 | 20.39 | NMIMR | STPH | DYM | SRR11444106 | Ghana |
| G32012 | 4234 | 76.5 | 17.7 | NMIMR | STPH | DYM | SRR11444402 | Ghana |
| G32015 | 3866 | 61.39 | 16.03 | NMIMR | STPH | DYM | SRR11444020 | Ghana |
| G32016 | 4016 | 85.12 | 16.82 | NMIMR | STPH | DYM | SRR11444001 | Ghana |
| G32017 | 2274 | 87.36 | 20.02 | NMIMR | STPH | DYM | SRR11444222 | Ghana |
| G32018 | 1393 | 90.44 | 17.85 | NMIMR | STPH | DYM | SRR11444362 | Ghana |
| G32749 | 1911 | 64.62 | 11.41 | NMIMR | STPH | DYM | SRR11444270 | Ghana |
| G32750 | 2973 | 66.26 | 11.48 | NMIMR | STPH | DYM | SRR11444126 | Ghana |
| G32752 | 3703 | 60.64 | 11.98 | NMIMR | STPH | DYM | SRR11444044 | Ghana |
| G32753 | 2428 | 63.26 | 10.4 | NMIMR | STPH | DYM | SRR11444205 | Ghana |
| G32754 | 3646 | 61.07 | 10.32 | NMIMR | STPH | DYM | SRR11444058 | Ghana |
| G32755 | 1512 | 58.47 | 10.82 | NMIMR | STPH | DYM | SRR11444346 | Ghana |
| G32756 | 1939 | 61.48 | 11.38 | NMIMR | STPH | DYM | SRR11444265 | Ghana |
| G32757 | 3677 | 50.91 | 12.42 | NMIMR | STPH | DYM | SRR11444050 | Ghana |
| G32758 | 1327 | 54.94 | 11.98 | NMIMR | STPH | DYM | SRR11444370 | Ghana |
| G32759 | 1530 | 59.51 | 12.03 | NMIMR | STPH | DYM | SRR11444345 | Ghana |
| G32760 | 1872 | 64.85 | 11.81 | NMIMR | STPH | DYM | SRR11444278 | Ghana |
| G32763 | 1668 | 59.41 | 9.62 | NMIMR | STPH | DYM | SRR11444323 | Ghana |
| G32764 | 3144 | 69.32 | 10.43 | NMIMR | STPH | DYM | SRR11444107 | Ghana |
| G32765 | 4206 | 64.3 | 11.56 | NMIMR | STPH | DYM | SRR11444409 | Ghana |
| G32766 | 3902 | 51.57 | 12.81 | NMIMR | STPH | DYM | SRR11444015 | Ghana |
| G32768 | 3798 | 60.45 | 13.52 | NMIMR | STPH | DYM | SRR11444030 | Ghana |
| G32769 | 1414 | 53.32 | 11.46 | NMIMR | STPH | DYM | SRR11444357 | Ghana |
| G32770 | 1032 | 64.24 | 10.86 | NMIMR | STPH | DYM | SRR11444336 | Ghana |
| G32771 | 3654 | 52.07 | 11.54 | NMIMR | STPH | DYM | SRR11444054 | Ghana |
| G32772 | 4210 | 52.56 | 11.37 | NMIMR | STPH | DYM | SRR11444408 | Ghana |
| G32773 | 4004 | 63.07 | 11.96 | NMIMR | STPH | DYM | SRR11444006 | Ghana |
| G32774 | 3678 | 58.92 | 11.14 | NMIMR | STPH | DYM | SRR11444049 | Ghana |
| G32775 | 1681 | 60.69 | 12 | NMIMR | STPH | DYM | SRR11444320 | Ghana |
| G32776 | 1581 | 69.78 | 12.9 | NMIMR | STPH | DYM | SRR11444339 | Ghana |
| G32777 | 2945 | 55.7 | 10.34 | NMIMR | STPH | DYM | SRR11444128 | Ghana |
| G32778 | 2559 | 60.91 | 13.05 | NMIMR | STPH | DYM | SRR11444180 | Ghana |
| G32779 | 1036 | 60.55 | 11.84 | NMIMR | STPH | DYM | SRR11444312 | Ghana |
| G32780 | 1448 | 58.84 | 10.02 | NMIMR | STPH | DYM | SRR11444352 | Ghana |
| G32781 | 3238 | 50.24 | 10.88 | NMIMR | STPH | DYM | SRR11444100 | Ghana |
| G32782 | 3431 | 76.28 | 15.62 | NMIMR | STPH | DYM | SRR11444075 | Ghana |
| G32784 | 1468 | 63.78 | 11.89 | NMIMR | STPH | DYM | SRR11444351 | Ghana |
| G32787 | 1275 | 60.56 | 10.51 | NMIMR | STPH | DYM | SRR11443979 | Ghana |
| G32788 | 1411 | 53.24 | 11.59 | NMIMR | STPH | DYM | SRR11444359 | Ghana |
| G32789 | 3586 | 58.73 | 11.63 | NMIMR | STPH | DYM | SRR11444065 | Ghana |
| G32790 | 1608 | 57.36 | 11.49 | NMIMR | STPH | DYM | SRR11444335 | Ghana |
| G32791 | 4188 | 63.31 | 18.34 | NMIMR | STPH | DYM | SRR11443981 | Ghana |
| G32793 | 1253 | 59.03 | 10.08 | NMIMR | STPH | DYM | SRR11444068 | Ghana |
| G32794 | 2904 | 54.25 | 10.58 | NMIMR | STPH | DYM | SRR11444132 | Ghana |
| G32795 | 3909 | 50.65 | 10.52 | NMIMR | STPH | DYM | SRR11444014 | Ghana |
| G32796 | 4319 | 56.01 | 10.82 | NMIMR | STPH | DYM | SRR11444386 | Ghana |
| G32797 | 3504 | 50.83 | 10.73 | NMIMR | STPH | DYM | SRR11444073 | Ghana |
| G32800 | 1709 | 59.3 | 9.79 | NMIMR | STPH | DYM | SRR11444308 | Ghana |
| G32801 | 1293 | 61.55 | 10.39 | NMIMR | STPH | DYM | SRR11444375 | Ghana |
| G32802 | 2686 | 64.41 | 10.25 | NMIMR | STPH | DYM | SRR11444161 | Ghana |
| G32803 | 2405 | 56.28 | 10.31 | NMIMR | STPH | DYM | SRR11444210 | Ghana |
| G32804 | 4306 | 53.83 | 11.1 | NMIMR | STPH | DYM | SRR11444387 | Ghana |
| G32805 | 2232 | 56.07 | 11.03 | NMIMR | STPH | DYM | SRR11444230 | Ghana |
| G32807 | 2472 | 63.71 | 11.13 | NMIMR | STPH | DYM | SRR11444193 | Ghana |
| G32808 | 2168 | 61.37 | 11.09 | NMIMR | STPH | DYM | SRR11444237 | Ghana |
| G32809 | 3756 | 59.73 | 9.93 | NMIMR | STPH | DYM | SRR11444037 | Ghana |
| G32811 | 2594 | 64.91 | 11.82 | NMIMR | STPH | DYM | SRR11444175 | Ghana |
| G32812 | 1606 | 57.58 | 11.67 | NMIMR | STPH | DYM | SRR11444338 | Ghana |
| G32813 | 2499 | 66.5 | 12.58 | NMIMR | STPH | DYM | SRR11444188 | Ghana |
| G32814 | 1674 | 66.27 | 11.94 | NMIMR | STPH | DYM | SRR11444322 | Ghana |
| G32815 | 1205 | 58.98 | 11.08 | NMIMR | STPH | DYM | SRR11444102 | Ghana |
| G32816 | 4243 | 63.14 | 10.69 | NMIMR | STPH | DYM | SRR11444400 | Ghana |
| G32818 | 2961 | 67.9 | 11.16 | NMIMR | STPH | DYM | SRR11444127 | Ghana |
| G32820 | 1895 | 60.44 | 11.08 | NMIMR | STPH | DYM | SRR11444274 | Ghana |
| G32821 | 2746 | 66.73 | 11.25 | NMIMR | STPH | DYM | SRR11444148 | Ghana |
| G32822 | 2506 | 65.86 | 11.61 | NMIMR | STPH | DYM | SRR11444186 | Ghana |
| G32823 | 2376 | 60.03 | 10.56 | NMIMR | STPH | DYM | SRR11444215 | Ghana |
| G32824 | 1660 | 57.96 | 9.68 | NMIMR | STPH | DYM | SRR11444326 | Ghana |
| G32826 | 2301 | 62.07 | 10.53 | NMIMR | STPH | DYM | SRR11444220 | Ghana |
| G32827 | 1193 | 56.83 | 10.49 | NMIMR | STPH | DYM | SRR11444135 | Ghana |
| G32828 | 2696 | 51.5 | 10.21 | NMIMR | STPH | DYM | SRR11444160 | Ghana |
| G32829 | 1886 | 53.83 | 10.59 | NMIMR | STPH | DYM | SRR11444276 | Ghana |
| G32830 | 4011 | 58.52 | 11.59 | NMIMR | STPH | DYM | SRR11444003 | Ghana |
| G32831 | 2606 | 63.18 | 12.12 | NMIMR | STPH | DYM | SRR11444173 | Ghana |
| G32832 | 4150 | 62.03 | 11.59 | NMIMR | STPH | DYM | SRR11443988 | Ghana |
| G32833 | 3686 | 58.95 | 10.65 | NMIMR | STPH | DYM | SRR11444047 | Ghana |
| G32834 | 3868 | 56.46 | 10.16 | NMIMR | STPH | DYM | SRR11444019 | Ghana |
| G32835 | 1950 | 57.36 | 11.21 | NMIMR | STPH | DYM | SRR11444264 | Ghana |
| G32837 | 1269 | 62.19 | 10.5 | NMIMR | STPH | DYM | SRR11444024 | Ghana |
| G32838 | 3018 | 54.3 | 11.2 | NMIMR | STPH | DYM | SRR11444120 | Ghana |
| G32839 | 3522 | 63.27 | 12.02 | NMIMR | STPH | DYM | SRR11444070 | Ghana |
| G32840 | 3273 | 57.17 | 10.75 | NMIMR | STPH | DYM | SRR11444094 | Ghana |
| G32841 | 2475 | 62.91 | 11.12 | NMIMR | STPH | DYM | SRR11444192 | Ghana |
| G32842 | 1147 | 51.79 | 9.6 | NMIMR | STPH | DYM | SRR11444168 | Ghana |
| G32843 | 1847 | 55.56 | 10.78 | NMIMR | STPH | DYM | SRR11444282 | Ghana |
| G32844 | 2470 | 53.26 | 10.01 | NMIMR | STPH | DYM | SRR11444194 | Ghana |
| G32845 | 1702 | 57.39 | 11.37 | NMIMR | STPH | DYM | SRR11444309 | Ghana |
| G32846 | 1099 | 63.35 | 12.49 | NMIMR | STPH | DYM | SRR11444213 | Ghana |
| G32847 | 1502 | 58.3 | 11.44 | NMIMR | STPH | DYM | SRR11444348 | Ghana |
| G32848 | 2546 | 60.27 | 11.06 | NMIMR | STPH | DYM | SRR11444181 | Ghana |
| G32849 | 3600 | 55.21 | 10.56 | NMIMR | STPH | DYM | SRR11444064 | Ghana |
| G32850 | 2627 | 59.7 | 11.23 | NMIMR | STPH | DYM | SRR11444169 | Ghana |
| G32851 | 3127 | 67.55 | 11.54 | NMIMR | STPH | DYM | SRR11444108 | Ghana |
| G32852 | 1683 | 59.49 | 9.94 | NMIMR | STPH | DYM | SRR11444319 | Ghana |
| G32853 | 1653 | 60.01 | 11.29 | NMIMR | STPH | DYM | SRR11444327 | Ghana |
| G32854 | 1547 | 54.6 | 11.12 | NMIMR | STPH | DYM | SRR11444343 | Ghana |
| G32855 | 1922 | 65.85 | 10.42 | NMIMR | STPH | DYM | SRR11444266 | Ghana |
| G32856 | 4295 | 65.18 | 11.98 | NMIMR | STPH | DYM | SRR11444388 | Ghana |
| G32858 | 2493 | 54.61 | 10.68 | NMIMR | STPH | DYM | SRR11444189 | Ghana |
| G32859 | 2503 | 65.91 | 11.41 | NMIMR | STPH | DYM | SRR11444187 | Ghana |
| G32860 | 2577 | 65.08 | 11.33 | NMIMR | STPH | DYM | SRR11444177 | Ghana |
| G32861 | 1201 | 58.12 | 11.23 | NMIMR | STPH | DYM | SRR11444113 | Ghana |
| G32862 | 4258 | 61.59 | 11.63 | NMIMR | STPH | DYM | SRR11444398 | Ghana |
| G32863 | 2652 | 60.18 | 10.97 | NMIMR | STPH | DYM | SRR11444166 | Ghana |
| G32867 | 1693 | 58.93 | 11.79 | NMIMR | STPH | DYM | SRR11444315 | Ghana |
| G32868 | 1643 | 51.3 | 10.34 | NMIMR | STPH | DYM | SRR11444329 | Ghana |
| G32869 | 1307 | 63.39 | 11.33 | NMIMR | STPH | DYM | SRR11444373 | Ghana |
| G32870 | 3520 | 66.16 | 12.24 | NMIMR | STPH | DYM | SRR11444071 | Ghana |
| G32871 | 2410 | 58.25 | 10.74 | NMIMR | STPH | DYM | SRR11444207 | Ghana |
| G32872 | 2077 | 57.85 | 12.12 | NMIMR | STPH | DYM | SRR11444245 | Ghana |
| G32873 | 1960 | 57.37 | 10.91 | NMIMR | STPH | DYM | SRR11444263 | Ghana |
| G32874 | 2866 | 52.27 | 10.39 | NMIMR | STPH | DYM | SRR11444137 | Ghana |
| G32875 | 2469 | 62.73 | 10.79 | NMIMR | STPH | DYM | SRR11444195 | Ghana |
| G32876 | 1633 | 57.9 | 11.72 | NMIMR | STPH | DYM | SRR11444332 | Ghana |
| G32877 | 2291 | 59.7 | 11.59 | NMIMR | STPH | DYM | SRR11444221 | Ghana |
| G32878 | 4415 | 56.75 | 12.32 | NMIMR | STPH | DYM | SRR11444378 | Ghana |
| G32879 | 1993 | 63.77 | 11.55 | NMIMR | STPH | DYM | SRR11444258 | Ghana |
| G32880 | 3713 | 58.23 | 12.1 | NMIMR | STPH | DYM | SRR11444042 | Ghana |
| G32881 | 2046 | 60.07 | 11.76 | NMIMR | STPH | DYM | SRR11444251 | Ghana |
| G32883 | 1377 | 58.86 | 12.23 | NMIMR | STPH | DYM | SRR11444364 | Ghana |
| G32884 | 2158 | 52.41 | 11.25 | NMIMR | STPH | DYM | SRR11444240 | Ghana |
| G32885 | 1470 | 54.07 | 12.22 | NMIMR | STPH | DYM | SRR11444350 | Ghana |
| G32955 | 4417 | 57 | 11.02 | NMIMR | STPH | DYM | SRR11444376 | Ghana |
| G32956 | 3412 | 100.51 | 27.97 | NMIMR | STPH | DYM | SRR11444078 | Ghana |
| G32957 | 3406 | 99.42 | 17.09 | NMIMR | STPH | DYM | SRR11444080 | Ghana |
| G32958 | 1837 | 91.55 | 16.91 | NMIMR | STPH | DYM | SRR11444284 | Ghana |
| G32959 | 1714 | 116.7 | 19.03 | NMIMR | STPH | DYM | SRR11444307 | Ghana |
| G32960 | 3990 | 104.52 | 18.15 | NMIMR | STPH | DYM | SRR11444009 | Ghana |
| G32961 | 4285 | 93.66 | 18.61 | NMIMR | STPH | DYM | SRR11444390 | Ghana |
| G32962 | 2152 | 92.03 | 17.71 | NMIMR | STPH | DYM | SRR11444241 | Ghana |
| G32963 | 4282 | 102.59 | 21.55 | NMIMR | STPH | DYM | SRR11444393 | Ghana |
| G32964 | 3425 | 121.12 | 19.71 | NMIMR | STPH | DYM | SRR11444076 | Ghana |
| G32965 | 1142 | 109.71 | 17.38 | NMIMR | STPH | DYM | SRR11444179 | Ghana |
| G32966 | 4199 | 99.61 | 17.03 | NMIMR | STPH | DYM | SRR11444411 | Ghana |
| G32967 | 3095 | 92.53 | 14.52 | NMIMR | STPH | DYM | SRR11444112 | Ghana |
| G32968 | 2837 | 107.48 | 19.26 | NMIMR | STPH | DYM | SRR11444139 | Ghana |
| G32969 | 2843 | 105.79 | 20.1 | NMIMR | STPH | DYM | SRR11444138 | Ghana |
| G32972 | 1717 | 141.43 | 22.11 | NMIMR | STPH | DYM | SRR11444304 | Ghana |
| G32975 | 4261 | 114.57 | 18.77 | NMIMR | STPH | DYM | SRR11444397 | Ghana |
| G32976 | 2685 | 104.04 | 17.94 | NMIMR | STPH | DYM | SRR11444162 | Ghana |
| G32977 | 2894 | 107.88 | 18.79 | NMIMR | STPH | DYM | SRR11444133 | Ghana |
| G32978 | 3300 | 122.23 | 20.66 | NMIMR | STPH | DYM | SRR11444089 | Ghana |
| G32980 | 4356 | 104.89 | 16.72 | NMIMR | STPH | DYM | SRR11444384 | Ghana |
| G32983 | 2791 | 69.82 | 9.84 | NMIMR | STPH | DYM | SRR11444143 | Ghana |
| G32984 | 2792 | 108.04 | 18.42 | NMIMR | STPH | DYM | SRR11444142 | Ghana |
| G32985 | 1440 | 98.18 | 17.28 | NMIMR | STPH | DYM | SRR11444354 | Ghana |
| G32986 | 3792 | 100.91 | 16.95 | NMIMR | STPH | DYM | SRR11444031 | Ghana |
| G32988 | 3579 | 130.37 | 22.52 | NMIMR | STPH | DYM | SRR11444067 | Ghana |
| G32989 | 1273 | 105.78 | 16.55 | NMIMR | STPH | DYM | SRR11443991 | Ghana |
| G32990 | 4152 | 105.38 | 17.98 | NMIMR | STPH | DYM | SRR11443987 | Ghana |
| G32991 | 2437 | 114.21 | 18.67 | NMIMR | STPH | DYM | SRR11444204 | Ghana |
| G32992 | 2933 | 92.4 | 16.45 | NMIMR | STPH | DYM | SRR11444130 | Ghana |
| G32994 | 1149 | 106.8 | 18.96 | NMIMR | STPH | DYM | SRR11444157 | Ghana |
| G32995 | 4066 | 100.29 | 18.09 | NMIMR | STPH | DYM | SRR11443995 | Ghana |
| G32996 | 1888 | 102.09 | 19 | NMIMR | STPH | DYM | SRR11444275 | Ghana |
| G32997 | 1427 | 103.23 | 16.56 | NMIMR | STPH | DYM | SRR11444356 | Ghana |
| G32998 | 4142 | 104 | 17.57 | NMIMR | STPH | DYM | SRR11443989 | Ghana |
| G32999 | 1070 | 98.44 | 15.31 | NMIMR | STPH | DYM | SRR11444268 | Ghana |
| G33000 | 1638 | 107.28 | 16.14 | NMIMR | STPH | DYM | SRR11444330 | Ghana |
| G33001 | 3517 | 92.02 | 16.65 | NMIMR | STPH | DYM | SRR11444072 | Ghana |
| G33002 | 1800 | 105.36 | 17.77 | NMIMR | STPH | DYM | SRR11444288 | Ghana |
| G33003 | 1354 | 102.67 | 19.09 | NMIMR | STPH | DYM | SRR11444366 | Ghana |
| G33004 | 3891 | 110.52 | 18.44 | NMIMR | STPH | DYM | SRR11444016 | Ghana |
| G33005 | 1914 | 85.07 | 14.38 | NMIMR | STPH | DYM | SRR11444267 | Ghana |
| G33006 | 1715 | 97.13 | 15.72 | NMIMR | STPH | DYM | SRR11444306 | Ghana |
| G33007 | 1979 | 105.76 | 16.83 | NMIMR | STPH | DYM | SRR11444260 | Ghana |
| G33008 | 3305 | 93.3 | 16.85 | NMIMR | STPH | DYM | SRR11444087 | Ghana |
| G33009 | 3685 | 112.46 | 19.9 | NMIMR | STPH | DYM | SRR11444048 | Ghana |
| G33010 | 3262 | 120.4 | 21.78 | NMIMR | STPH | DYM | SRR11444096 | Ghana |
| G33011 | 2044 | 116.09 | 21.07 | NMIMR | STPH | DYM | SRR11444252 | Ghana |
| G33012 | 3631 | 117.94 | 20.06 | NMIMR | STPH | DYM | SRR11444061 | Ghana |
| G33013 | 1084 | 92.46 | 14.52 | NMIMR | STPH | DYM | SRR11444235 | Ghana |
| G33014 | 2204 | 109.62 | 17.66 | NMIMR | STPH | DYM | SRR11444233 | Ghana |
| G33015 | 2733 | 117.93 | 19.03 | NMIMR | STPH | DYM | SRR11444151 | Ghana |
| G33016 | 2939 | 103.45 | 16.19 | NMIMR | STPH | DYM | SRR11444129 | Ghana |
| G33017 | 2705 | 99.99 | 17.27 | NMIMR | STPH | DYM | SRR11444156 | Ghana |
| G33018 | 3877 | 132.63 | 18.79 | NMIMR | STPH | DYM | SRR11444018 | Ghana |
| G33019 | 4093 | 106.23 | 18.7 | NMIMR | STPH | DYM | SRR11443990 | Ghana |
| G33020 | 3676 | 135.14 | 22.79 | NMIMR | STPH | DYM | SRR11444051 | Ghana |
| G33021 | 4193 | 123.43 | 20.1 | NMIMR | STPH | DYM | SRR11444413 | Ghana |
| G33022 | 2227 | 109.69 | 17.36 | NMIMR | STPH | DYM | SRR11444231 | Ghana |
| G33023 | 3307 | 108.51 | 18.48 | NMIMR | STPH | DYM | SRR11444086 | Ghana |
| G33024 | 3062 | 116.3 | 16.32 | NMIMR | STPH | DYM | SRR11444115 | Ghana |
| G33025 | 2700 | 108.26 | 18.47 | NMIMR | STPH | DYM | SRR11444159 | Ghana |
| G33027 | 3046 | 116.76 | 20.19 | NMIMR | STPH | DYM | SRR11444117 | Ghana |
| G33028 | 4168 | 115.08 | 17.73 | NMIMR | STPH | DYM | SRR11443984 | Ghana |
| G33029 | 1259 | 88.31 | 15.41 | NMIMR | STPH | DYM | SRR11444046 | Ghana |
| G33030 | 1401 | 104.52 | 19.21 | NMIMR | STPH | DYM | SRR11444361 | Ghana |
| G33031 | 1318 | 122.02 | 17.52 | NMIMR | STPH | DYM | SRR11444371 | Ghana |
| G33032 | 2624 | 114.78 | 18.56 | NMIMR | STPH | DYM | SRR11444171 | Ghana |
| G33033 | 1794 | 119.1 | 20.56 | NMIMR | STPH | DYM | SRR11444292 | Ghana |
| G33034 | 2701 | 123.25 | 21.54 | NMIMR | STPH | DYM | SRR11444158 | Ghana |
| G33035 | 3565 | 97.47 | 16.82 | NMIMR | STPH | DYM | SRR11444069 | Ghana |
| G33036 | 1028 | 109 | 19.84 | NMIMR | STPH | DYM | SRR11444358 | Ghana |
| G33037 | 1748 | 117.67 | 20.74 | NMIMR | STPH | DYM | SRR11444298 | Ghana |
| G33038 | 3860 | 114.7 | 19.33 | NMIMR | STPH | DYM | SRR11444022 | Ghana |
| G33039 | 4257 | 125.09 | 20.06 | NMIMR | STPH | DYM | SRR11444399 | Ghana |
| G33040 | 1628 | 100.82 | 15.75 | NMIMR | STPH | DYM | SRR11444333 | Ghana |
| G33041 | 2074 | 46.22 | 9.96 | NMIMR | STPH | DYM | SRR11444248 | Ghana |
| G33042 | 2681 | 109.82 | 19.4 | NMIMR | STPH | DYM | SRR11444163 | Ghana |
| G33043 | 4360 | 118.72 | 19.68 | NMIMR | STPH | DYM | SRR11444383 | Ghana |
| G33044 | 3739 | 102.11 | 17.48 | NMIMR | STPH | DYM | SRR11444041 | Ghana |
| G33045 | 1873 | 127.85 | 18.58 | NMIMR | STPH | DYM | SRR11444277 | Ghana |
| G33046 | 2638 | 111.77 | 16.65 | NMIMR | STPH | DYM | SRR11444167 | Ghana |
| G33047 | 1549 | 128.68 | 21.29 | NMIMR | STPH | DYM | SRR11444342 | Ghana |
| G33049 | 3000 | 115.54 | 17.84 | NMIMR | STPH | DYM | SRR11444123 | Ghana |
| G33050 | 2571 | 120.09 | 21.05 | NMIMR | STPH | DYM | SRR11444178 | Ghana |
| G33051 | 1770 | 67.63 | 13.77 | NMIMR | STPH | DYM | SRR11444294 | Ghana |
| G33053 | 3010 | 65.24 | 11.48 | NMIMR | STPH | DYM | SRR11444122 | Ghana |
| G33054 | 1607 | 57.71 | 9.5 | NMIMR | STPH | DYM | SRR11444337 | Ghana |
| G33055 | 4059 | 61.89 | 10.95 | NMIMR | STPH | DYM | SRR11443996 | Ghana |
| G33056 | 3344 | 67.7 | 11.95 | NMIMR | STPH | DYM | SRR11444085 | Ghana |
| G33057 | 2320 | 71.45 | 11.69 | NMIMR | STPH | DYM | SRR11444218 | Ghana |
| G33058 | 1476 | 62.07 | 11.24 | NMIMR | STPH | DYM | SRR11444349 | Ghana |
| G33059 | 3694 | 52.11 | 9.99 | NMIMR | STPH | DYM | SRR11444045 | Ghana |
| G33060 | 3938 | 50.28 | 10.57 | NMIMR | STPH | DYM | SRR11444012 | Ghana |
| G33061 | 2257 | 59.39 | 10.64 | NMIMR | STPH | DYM | SRR11444227 | Ghana |
| G33062 | 3664 | 51.75 | 8.46 | NMIMR | STPH | DYM | SRR11444053 | Ghana |
| G33064 | 2875 | 52.75 | 9.94 | NMIMR | STPH | DYM | SRR11444136 | Ghana |
| G33065 | 1716 | 60.98 | 10.16 | NMIMR | STPH | DYM | SRR11444305 | Ghana |
| G33066 | 1018 | 45.98 | 8.69 | NMIMR | STPH | DYM | SRR11444091 | Ghana |
| G33067 | 1854 | 59.36 | 10.62 | NMIMR | STPH | DYM | SRR11444281 | Ghana |
| G33068 | 1034 | 61.68 | 10.96 | NMIMR | STPH | DYM | SRR11444325 | Ghana |
| G33069 | 2260 | 49.11 | 9.23 | NMIMR | STPH | DYM | SRR11444226 | Ghana |
| G33070 | 4078 | 56.76 | 8.74 | NMIMR | STPH | DYM | SRR11443993 | Ghana |
| G33071 | 2477 | 53.7 | 9.55 | NMIMR | STPH | DYM | SRR11444191 | Ghana |
| G33072 | 3647 | 48.75 | 8.44 | NMIMR | STPH | DYM | SRR11444056 | Ghana |
| G33073 | 4270 | 68.09 | 11.79 | NMIMR | STPH | DYM | SRR11444396 | Ghana |
| G33074 | 3787 | 51.07 | 10.11 | NMIMR | STPH | DYM | SRR11444032 | Ghana |
| G33075 | 1073 | 58.51 | 10.74 | NMIMR | STPH | DYM | SRR11444257 | Ghana |
| G33076 | 1350 | 62.85 | 11 | NMIMR | STPH | DYM | SRR11444367 | Ghana |
| G33077 | 1008 | 31.89 | 7.05 | NMIMR | STPH | DYM | SRR11444314 | Ghana |
| G33078 | 2736 | 56.58 | 9.74 | NMIMR | STPH | DYM | SRR11444149 | Ghana |
| G33079 | 4006 | 32.93 | 6.88 | NMIMR | STPH | DYM | SRR11444005 | Ghana |
| G33080 | 4233 | 54.64 | 9.27 | NMIMR | STPH | DYM | SRR11444404 | Ghana |
| G33082 | 1023 | 52.55 | 9.97 | NMIMR | STPH | DYM | SRR11444369 | Ghana |
| G33083 | 1899 | 60.94 | 11.66 | NMIMR | STPH | DYM | SRR11444273 | Ghana |
| G33084 | 2538 | 55.38 | 9.35 | NMIMR | STPH | DYM | SRR11444183 | Ghana |
| G33085 | 2461 | 60.75 | 10.66 | NMIMR | STPH | DYM | SRR11444197 | Ghana |
| G33087 | 3649 | 59.88 | 10.22 | NMIMR | STPH | DYM | SRR11444055 | Ghana |
| G33088 | 3158 | 56.95 | 9.62 | NMIMR | STPH | DYM | SRR11444105 | Ghana |
| G33089 | 2254 | 59.05 | 10.55 | NMIMR | STPH | DYM | SRR11444228 | Ghana |
| G33090 | 3818 | 52.46 | 9.66 | NMIMR | STPH | DYM | SRR11444027 | Ghana |
| G33091 | 4036 | 47.24 | 9.76 | NMIMR | STPH | DYM | SRR11443999 | Ghana |
| G33092 | 2078 | 48.09 | 9.88 | NMIMR | STPH | DYM | SRR11444244 | Ghana |
| G33094 | 3582 | 49.44 | 9.22 | NMIMR | STPH | DYM | SRR11444066 | Ghana |
| G33096 | 2793 | 43.63 | 8.82 | NMIMR | STPH | DYM | SRR11444141 | Ghana |
| G33097 | 3125 | 51.16 | 9.46 | NMIMR | STPH | DYM | SRR11444109 | Ghana |
| G33099 | 3303 | 67.41 | 12.22 | NMIMR | STPH | DYM | SRR11444088 | Ghana |
| G33100 | 1100 | 56.81 | 9.84 | NMIMR | STPH | DYM | SRR11444201 | Ghana |
| G33101 | 4211 | 58.08 | 10.44 | NMIMR | STPH | DYM | SRR11444407 | Ghana |
| G33102 | 3635 | 63.68 | 10.97 | NMIMR | STPH | DYM | SRR11444059 | Ghana |
| G33103 | 1913 | 54.47 | 9.67 | NMIMR | STPH | DYM | SRR11444269 | Ghana |
| G33104 | 1725 | 49.01 | 9.29 | NMIMR | STPH | DYM | SRR11444302 | Ghana |
| G33106 | 1689 | 53.54 | 10.43 | NMIMR | STPH | DYM | SRR11444316 | Ghana |
| G33107 | 3766 | 42.84 | 9.06 | NMIMR | STPH | DYM | SRR11444034 | Ghana |
| G33108 | 4416 | 48.23 | 9.88 | NMIMR | STPH | DYM | SRR11444377 | Ghana |
| G33110 | 4186 | 47.45 | 9.2 | NMIMR | STPH | DYM | SRR11443982 | Ghana |
| G33111 | 3015 | 54.25 | 9.13 | NMIMR | STPH | DYM | SRR11444121 | Ghana |
| G33112 | 2448 | 45.22 | 9.04 | NMIMR | STPH | DYM | SRR11444199 | Ghana |
| G33113 | 3271 | 47.96 | 9.74 | NMIMR | STPH | DYM | SRR11444095 | Ghana |
| G33114 | 4031 | 44.98 | 8.87 | NMIMR | STPH | DYM | SRR11444000 | Ghana |
| G33115 | 1101 | 56.62 | 10.71 | NMIMR | STPH | DYM | SRR11444190 | Ghana |
| G33116 | 4077 | 57.45 | 9.56 | NMIMR | STPH | DYM | SRR11443994 | Ghana |
| G33117 | 2589 | 57.96 | 8.94 | NMIMR | STPH | DYM | SRR11444176 | Ghana |
| G33118 | 2099 | 50.74 | 8.75 | NMIMR | STPH | DYM | SRR11444243 | Ghana |
| G33120 | 3122 | 61.29 | 10.49 | NMIMR | STPH | DYM | SRR11444110 | Ghana |
| G33126 | 2161 | 58.37 | 10.8 | NMIMR | STPH | DYM | SRR11444239 | Ghana |
| G33127 | 2524 | 61.91 | 11.35 | NMIMR | STPH | DYM | SRR11444185 | Ghana |
| G33130 | 2024 | 66.73 | 11.5 | NMIMR | STPH | DYM | SRR11444255 | Ghana |
| G33136 | 1254 | 66.01 | 12.62 | NMIMR | STPH | DYM | SRR11444057 | Ghana |
| G33140 | 3060 | 66.94 | 13.15 | NMIMR | STPH | DYM | SRR11444116 | Ghana |
| G33142 | 4165 | 44.69 | 12.03 | NMIMR | STPH | DYM | SRR11443985 | Ghana |
| G33143 | 4080 | 61.47 | 13.19 | NMIMR | STPH | DYM | SRR11443992 | Ghana |
| G33144 | 4380 | 54.26 | 10.39 | NMIMR | STPH | DYM | SRR11444382 | Ghana |
| G33145 | 4204 | 67.41 | 12.1 | NMIMR | STPH | DYM | SRR11444410 | Ghana |
| G33148 | 1806 | 15.19 | 4.4 | NMIMR | STPH | DYM | SRR11444287 | Ghana |
| G33149 | 4281 | 54.27 | 10.71 | NMIMR | STPH | DYM | SRR11444394 | Ghana |
| G33151 | 3246 | 59.5 | 10.39 | NMIMR | STPH | DYM | SRR11444097 | Ghana |
| G33152 | 3365 | 61.07 | 10.5 | NMIMR | STPH | DYM | SRR11444082 | Ghana |
| G33157 | 3161 | 56.13 | 10.27 | NMIMR | STPH | DYM | SRR11444104 | Ghana |
| G33158 | 1298 | 55.05 | 9.02 | NMIMR | STPH | DYM | SRR11444374 | Ghana |
| G33159 | 4284 | 59.17 | 11.24 | NMIMR | STPH | DYM | SRR11444391 | Ghana |
| G33161 | 3194 | 59.83 | 11.95 | NMIMR | STPH | DYM | SRR11444103 | Ghana |
| G33162 | 2075 | 58.64 | 11.43 | NMIMR | STPH | DYM | SRR11444247 | Ghana |
| G33163 | 1694 | 57.15 | 9.6 | NMIMR | STPH | DYM | SRR11444311 | Ghana |
| G33165 | 3800 | 61.07 | 10.99 | NMIMR | STPH | DYM | SRR11444029 | Ghana |
| G33168 | 3863 | 70.69 | 10.79 | NMIMR | STPH | DYM | SRR11444021 | Ghana |
| G33169 | 3829 | 70.85 | 12.47 | NMIMR | STPH | DYM | SRR11444025 | Ghana |
| G33171 | 2387 | 59.12 | 9.71 | NMIMR | STPH | DYM | SRR11444214 | Ghana |
| G33173 | 1392 | 63.92 | 9.51 | NMIMR | STPH | DYM | SRR11444363 | Ghana |
| G33174 | 3969 | 39.64 | 7.01 | NMIMR | STPH | DYM | SRR11444010 | Ghana |

**Supplementary table 2.** Clustering analysis stratified by location of residence for large clusters

| **Residential district** | **all large clusters n(%)** | **WGSC-5 n(%)** |
| --- | --- | --- |
| **Ablekuma** | 37 (25.52) | 19 (24.68) |
| **Adenta Municipal** | 1 (0.69) | - |
| **Akwapim South** | 1 (0.69) | 1 (1.3) |
| **Ashiedu Keteke** | 11 (7.59) | 8 (10.39) |
| **Ashaiman Municipal** | 1 (0.69) | - |
| **Awutu Senya** | 1 (0.69) | 1 (1.3) |
| **Ayawaso** | 23 (15.86) | 8 (10.39) |
| **Ga Central** | 2 (1.38) | - |
| **Ga East** | 4 (2.76) | 2 (2.6) |
| **Ga South** | 3 (2.07) | 3 (3.9) |
| **Ga West** | 4 (2.76) | 1 (1.3) |
| **Kpeshie** | 12 (8.28) | 9 (11.69) |
| **La-Nkwantanang Madina Municipal** | 1 (0.69) | 1 (1.3) |
| **Mamprusi East** | 1 (0.69) | 1 (1.3) |
| **Northern Ghana** | 1 (0.69) | 1 (1.3) |
| **Okaikoi** | 16 (11.03) | 12 (15.58) |
| **Osu Klottey** | 3 (2.07) | 1 (1.3) |
| **Southern Ghana** | 21 (14.48) | 9 (11.69) |
| **Tamale Metropolis** | 1 (0.69) | - |
| **Tema Municipal** | 1 (0.69) | - |
| **Total** | 145 (100) | 77 (100) |
